# Tamoxifen induces PI3K activation in uterine cancer

**DOI:** 10.1038/s41588-025-02308-w

**Published:** 2025-08-22

**Authors:** Kirsten Kübler, Agostina Nardone, Shankara Anand, Daniel Gurevich, Jianjiong Gao, Marjolein Droog, Francisco Hermida-Prado, Tara Akhshi, Ariel Feiglin, Avery S. Feit, Gabriella Cohen Feit, Gwen Dackus, Matthew Pun, Yanan Kuang, Justin Cha, Mendy Miller, Sebastian Gregoricchio, Mirthe Lanfermeijer, Sten Cornelissen, William J. Gibson, Cloud P. Paweletz, Eliezer M. Van Allen, Flora E. van Leeuwen, Petra M. Nederlof, Quang-Dé Nguyen, Marian J. E. Mourits, Milan Radovich, Ignaty Leshchiner, Chip Stewart, Ursula A. Matulonis, Wilbert Zwart, Yosef E. Maruvka, Gad Getz, Rinath Jeselsohn

**Affiliations:** 1 Broad Institute of MIT and Harvard, Cambridge, MA USA; 2 Krantz Family Center for Cancer Research, Massachusetts General Hospital, Charlestown, MA USA; 3 Harvard Medical School, Boston, MA USA; 4 Berlin Institute of Health at Charité–Universitätsmedizin Berlin, Berlin, Germany; 5 Department of Hematology, Oncology and Cancer Immunology, Charité–Universitätsmedizin Berlin, corporate member of Freie Universität Berlin and Humboldt-Universität zu Berlin, Berlin, Germany; 6 German Cancer Consortium (DKTK), Partner Site Berlin and German Cancer Research Center (DKFZ), Heidelberg, Germany; 7 Center for Functional Cancer Epigenetics, Dana-Farber Cancer Institute, Boston, MA USA; 8 Biotechnology and Food Engineering, Technion, Haifa, Israel; 9 Lokey Center for Life Science and Engineering, Technion, Haifa, Israel; 10 Caris Life Sciences, Phoenix, AZ USA; 11 Division of Oncogenomics, Oncode Institute, Netherlands Cancer Institute, Amsterdam, the Netherlands; 12 Department of Biomedical Informatics, Harvard Medical School, Boston, MA USA; 13 Division of Molecular Pathology, Netherlands Cancer Institute, Amsterdam, the Netherlands; 14 Department of Pathology, Radboud University Medical Center, Nijmegen, the Netherlands; 15 Belfer Center for Applied Cancer Science, Dana-Farber Cancer Institute, Boston, MA USA; 16 Department of Laboratory Medicine, Netherlands Cancer Institute, Amsterdam, the Netherlands; 17 Core Facility Molecular Pathology & Biobanking, Netherlands Cancer Institute, Amsterdam, the Netherlands; 18 Center for Cancer Precision Medicine, Dana-Farber Cancer Institute, Boston, MA USA; 19 Department of Epidemiology, Netherlands Cancer Institute, Amsterdam, the Netherlands; 20 Department of Pathology, Netherlands Cancer Institute, Amsterdam, the Netherlands; 21 Lurie Family Imaging Center, Center for Biomedical Imaging in Oncology, Dana-Farber Cancer Institute, Boston, MA USA; 22 Department of Gynecological Oncology, University Medical Center Groningen, University of Groningen, Groningen, the Netherlands; 23 Department of Medicine, Boston University School of Medicine, Boston, MA USA; 24 The Susan F. Smith Center for Women’s Cancers, Dana-Farber Cancer Institute, Boston, MA USA; 25 Division of Gynecologic Oncology, Dana-Farber Cancer Institute, Boston, MA USA; 26 Laboratory of Chemical Biology and Institute for Complex Molecular Systems, Department of Biomedical Engineering, Eindhoven University of Technology, Eindhoven, the Netherlands; 27 Department of Pathology, Massachusetts General Hospital, Boston, MA USA; 28 Present Address: Harvard Medical School, Boston, MA USA

**Keywords:** Endometrial cancer, Breast cancer

## Abstract

Mutagenic processes and clonal selection contribute to the development of therapy-associated secondary neoplasms, a known complication of cancer treatment. The association between tamoxifen therapy and secondary uterine cancers is uncommon but well established; however, the genetic mechanisms underlying tamoxifen-driven tumorigenesis remain unclear. We find that oncogenic *PIK3CA* mutations, common in spontaneously arising estrogen-associated de novo uterine cancer, are significantly less frequent in tamoxifen-associated tumors. In vivo, tamoxifen-induced estrogen receptor stimulation activates phosphoinositide 3-kinase (PI3K) signaling in normal mouse uterine tissue, potentially eliminating the selective benefit of PI3K-activating mutations in tamoxifen-associated uterine cancer. Together, we present a unique pathway of therapy-associated carcinogenesis in which tamoxifen-induced activation of the PI3K pathway acts as a non-genetic driver event, contributing to the multistep model of uterine carcinogenesis. While this PI3K mechanism is specific to tamoxifen-associated uterine cancer, the concept of treatment-induced signaling events may have broader applicability to other routes of tumorigenesis.

## Abstract (web summary)

Sequencing analysis of tamoxifen-associated uterine cancers and further in vivo analyses suggest that the drug tamoxifen can activate the PI3K pathway in the absence of oncogenic mutations.

## Main

Therapy-related secondary malignancies associated with certain cytotoxic drugs or radiotherapy are relatively uncommon. Mechanistically, such secondary neoplasms are attributed to clonal selection of preexisting mutations or therapy-induced mutagenesis^1^. Whether similar mechanisms also contribute to cancer evolution after hormonal therapy has remained controversial, particularly in the context of tamoxifen use^2–7^.

Tamoxifen was the first endocrine drug approved for treating estrogen receptor (ER)-positive breast cancer^8,9^ and as a preventive drug in women with high risk of developing breast cancer^10^. Although estrogen-reducing aromatase inhibitors have superior outcome in the adjuvant setting^11^, tamoxifen still has a clear benefit in reducing risk of recurrence and death from breast cancer and remains a standard endocrine treatment option in premenopausal and postmenopausal women with early-stage ER^+^ disease^12,13^. One serious drawback of tamoxifen therapy is an association with increased risk of uterine cancer (UC): randomized clinical trials and large observational studies found a twofold to sevenfold increased risk 2–5 years after tamoxifen treatment either for breast cancer^14–18^ or for prevention^19,20^. Extended tamoxifen use of 10 versus 5 years correlated with an approximate twofold further increase in the risk of tamoxifen-associated UC (TA-UC)^21^, underscoring the link between tamoxifen and UC.

Tamoxifen is a selective ER modulator. In breast tissue, it functions as an ER antagonist; in the uterus, it has ER-agonistic activity stemming from the recruitment of ER coactivators rather than co-repressors^22^. The pro-proliferative effect of tamoxifen in the uterus is well established to be ER dependent^23,24^. However, whether this ER-agonistic effect is the key driver of oncogenesis in TA-UC remains unclear. Although tamoxifen has been reported to be mutagenic in the rat liver^25^, whether similar mutagenic effects occur in human uterine tissue remains controversial^26^. A previous study, limited in technological scope, did not find TA-UC-specific genomic changes^27^. Here, we extended the genomic profiling of TA-UCs to whole-exome sequencing (WES), allowing us to study a larger number and broader variety of genomic events. WES analysis and subsequent in vivo modeling in mice revealed a unique cancer development mechanism, an understanding that may have implications for counseling and risk-reducing interventions in tamoxifen-treated patients at high risk for UC as well as relevance to other therapy-related secondary cancers.

## Results

### No evidence of tamoxifen-induced mutagenesis

**Fig. 1:**
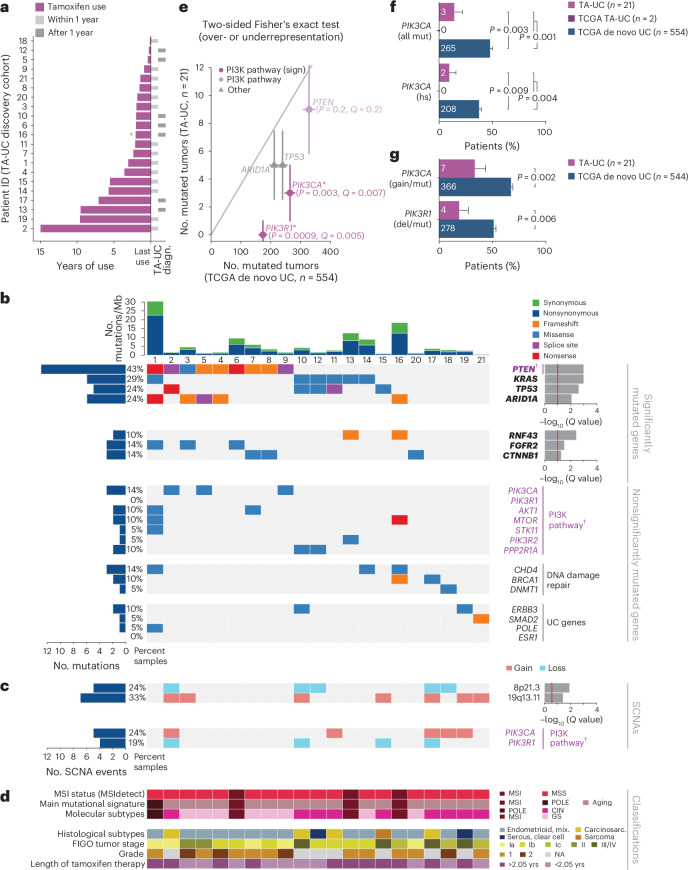
Reduced frequency of PI3K pathway mutations in TA-UC. **a**, Time course for each patient shows duration of tamoxifen treatment (colored bars) and periods of UC diagnosis (diagn., gray bars); crossed dagger indicates treatment for at least 2 years, but exact duration is unknown. **b**, Plot of mutational features for TA-UCs from the discovery cohort, ordered by significantly mutated genes. From top to bottom, subpanels depict number (no.) of mutations per megabase (Mb), sample identifiers, significantly mutated genes (bold; red line, *Q* < 0.1; top, unrestricted hypothesis testing; bottom, restricted hypothesis testing of known UC driver genes) and nonsignificantly mutated cancer genes (PI3K pathway genes are in violet and annotated with a dagger). **c**, Plot of SCNAs ordered as in **b**; top, significant SCNAs (red line, *Q* < 0.25, from GISTIC); bottom, nonsignificant SCNAs in the PI3K pathway (violet and annotated with a dagger). **d**, Plot of molecular classifications and mutational processes (MSI, microsatellite instability; MSS, microsatellite stable; CIN, chromosomal instability; GS, genomically stable; POLE, polymerase ε), clinical annotations (mix., mixed; carcinosarc., carcinosarcoma; FIGO, International Federation of Gynecology and Obstetrics; NA, not available) and median length of tamoxifen use in years (yrs); samples ordered as in **b**. **e**, UC driver genes powered to detect differences (higher or lower) in mutation frequencies between TCGA de novo UC and TA-UC sample sets (*P*-value threshold for statistical power analysis at <0.05 after Bonferroni correction for the 49 significant driver genes in de novo UC). Genes are colored by pathway; gray line indicates equal frequencies in both cohorts; data points represent number of mutated tumors; error bars reflect Poisson-based s.d. estimate. Significance analysis by two-sided BH-corrected Fisher’s exact test (*Q* values added for all *Q* < 0.1 and/or PI3K pathway genes; * and sign denote significance). **f**, Bar plot of all (top, all mut) and hotspot (hs, bottom) *PIK3CA* mutations; bars represent mutation frequencies; error bars reflect s.d. from the β-distribution; significance analysis by two-sided Fisher’s exact test; numbers in bars indicate mutated tumor count per group. **g**, Bar plot of PI3K pathway alterations including SNVs (mut) and SCNAs (gain or deletion (del)); only TCGA tumors with both data types were considered; genes altered by either type were counted once per tumor; bars represent mutation frequencies; error bars reflect s.d. from the β-distribution; significance analysis by two-sided Fisher’s exact test; numbers in bars indicate mutated tumor count per group.

**Extended Data Fig. 1:**
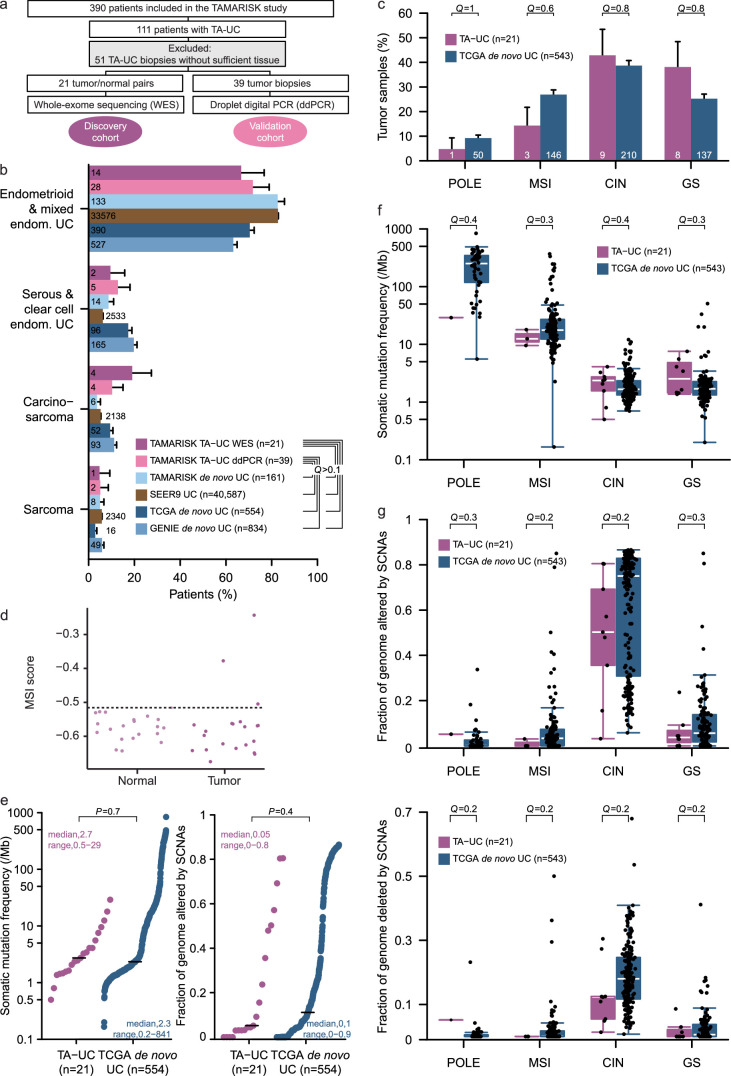
Absence of tamoxifen-induced mutagenesis in TA-UC. (**a**) CONSORT flow diagram depicts allocation of Netherlands Cancer Institute (NKI) patients from the TAMARISK study for our analysis. (**b**) Bar plot of uterine cancer (UC) cohorts with and without history of tamoxifen (TA); bars represent histological type frequencies (endom., endometrial); error bars reflect standard deviation from the β-distribution; significance analysis by two-sided Fisher’s exact test with Benjamini-Hochberg procedure; numbers in/above bars indicate tumor count per group. (**c**) Bar plot of TA-UC and de novo UC cases (excluding 7 endometroid and 4 serous endometrial TCGA tumors due to lack of annotation); bars represent molecular subtype frequencies; error bars reflect standard deviation from the β-distribution; numbers in bars indicate tumor count per group. Significance analysis by two-sided Fisher’s exact test with Benjamini-Hochberg procedure (CIN, chromosomal instable; GS, genomically stable; MSI, microsatellite instability; POLE, polymerase ε). (**d**) MSI scores for each TA-UC sample (dots), generated by MSIDetect (see Methods); corresponding normal samples served as controls; tumors with a higher score than in the normal were classified as MSI cases. (**e**) Number of non-synonymous mutations per exome (mutations/Megabase, left) and fraction of chromosomal regions affected by ABSOLUTE somatic copy number alterations (SCNAs) out of all measured regions (right); dots represent single samples; horizontal lines indicate group medians. Significance analysis by two-sided Wilcoxon test. (**f**) Number of non-synonymous mutations grouped by molecular subtype as in **c**. Individual data points (black) overlay summary statistic boxplots; horizontal center lines indicate median; boxes span the interquartile range (IQR, 25th to 75th percentile); whiskers extend to the most extreme values within 1.5×IQR. Significance analysis by two-sided Wilcoxon test with Benjamini-Hochberg procedure. (**g**) Number of chromosomal regions affected by somatic copy number alterations (that is, amplifications and deletions; top) or deletions only (bottom), grouped by molecular subtypes as in **c**. Individual data points (black) overlay summary statistic boxplots; horizontal center lines indicate median; boxes span the interquartile range (IQR, 25th to 75th percentile); whiskers extend to the most extreme values within 1.5×IQR. Significance analysis by two-sided Wilcoxon test with Benjamini-Hochberg procedure.

To determine whether TA-UC is molecularly distinct from spontaneously arising de novo UC (that is, not associated with tamoxifen), we performed WES on 21 TA-UCs from the ‘Tamoxifen Associated Malignancies: Aspects of Risk’ (TAMARISK) study^28^ (discovery cohort; Fig. 1a, Supplementary Table 1 and Extended Data Fig. 1a) and compared their histological types to various de novo UC cohorts (Surveillance, Epidemiology, and End Results 9 (SEER9), TAMARISK^28^, TCGA^29–31^, Genomics Evidence Neoplasia Information Exchange (GENIE)^32^). Our analysis revealed no significant differences after correcting for multiple hypotheses (all *Q* > 0.1, Benjamini–Hochberg (BH)-corrected Fisher’s exact test; Extended Data Fig. 1b and Supplementary Table 2). Similarly, the molecular subtypes in TA-UC closely matched those in de novo UC from TCGA^29^ (all *Q* > 0.5; Extended Data Fig. 1c,d, Supplementary Table 2 and Supplementary Note 1). These findings allow for downstream comparison of genomic alterations between TA-UC and de novo UC, independent of subtype.

**Extended Data Fig. 2:**
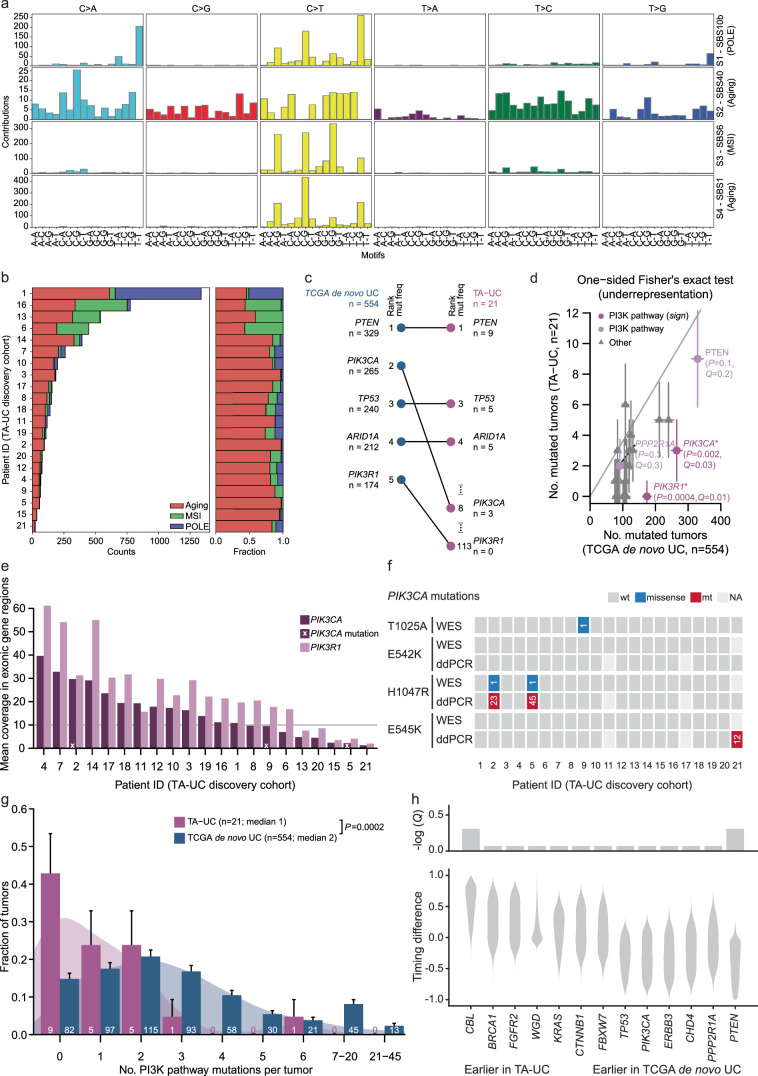
Mutational alterations in TA-UC. (**a**) Mutational matrix of TA-UC (discovery cohort) decomposed into four signatures; colors represent the six base substitution types (top y-axis), further stratified by 5’ and 3’ flanking bases (bottom y-axis). Patterns were matched to COSMIC reference signatures; known etiologies are shown on the right. (**b**) Mutational signature activity per sample, shown as count (left) and fraction (right) of mutations attributed to each signature (color-coded; identified as in **a**). (**c**) Rank order of UC driver genes powered to detect differences (higher or lower) in mutation frequencies (mut freq) between TCGA de novo UC and TA-UC (discovery cohort); lines connect gene ranks between cohorts. (**d**) UC driver genes powered to detect lower mutation frequencies in TA-UC compared to de novo UC (*P*-value threshold for statistical power analysis at <0.05, genes mutated in at least 76 de novo UC samples can potentially be considered significantly less mutated in TA-UC). Genes are colored by pathway. Gray line indicates equal frequencies; data points represent number (no) of mutated tumors; error bars reflect Poisson-based standard deviation estimate. Significance analysis by one-sided Fisher’s exact test with Benjamini-Hochberg procedure (*Q*-values added for all *Q* < 0.1 and/or PI3K pathway genes; * and *sign* denote significance). (**e**) Bar plot of mean gene coverage across samples, ordered high to low; gray line indicates the low-coverage threshold; white crosses indicate presence of a mutation. (**f**) Integrated plot of *PIK3CA* mutations in TA-UC samples detected by whole-exome sequencing (WES; blue; cancer cell fraction [CCF] shown) and droplet digital PCR (ddPCR; red; variant allele frequency [VAF; %] shown), ordered top to bottom by protein change (NA, not available). (**g**) Density histogram showing fraction of tumors grouped by number (no.) of mutations in key PI3K pathway genes per sample; error bars reflect standard deviation from the β-distribution; significance analysis by two-sided Wilcoxon test; numbers in bars indicate mutated tumor counts per group. (**h**) Violin plots showing timing differences of early clonal driver mutations between TA-UC and TCGA de novo UC; significance values from permutation tests with Benjamini-Hochberg procedure.

We next analyzed frequencies of genomic alterations to test for tamoxifen-related mutagenesis. Tamoxifen did not increase the mutational burden (median number of mutations per Mb, 2.7 in TA-UC versus 2.3 in de novo UC; *P* = 0.7, Wilcoxon test) or the genomic fraction affected by somatic copy number alterations (SCNAs; median of 0.05 versus 0.1, *P* = 0.4; Extended Data Fig. 1e), even after accounting for molecular subtypes (Extended Data Fig. 1f,g, Supplementary Table 2 and Supplementary Note 1). Similarly, the duration of tamoxifen treatment was unrelated to mutational (*r* = 0.07, Pearson correlation coefficient, *P* = 0.8) and SCNA burden (*r* = 0.3, *P* = 0.2). Mutational signatures can also reveal the mutagenic mechanisms of carcinogens^33^. While de novo signature discovery did not identify a tamoxifen-specific mutational signature, previously described signatures were detected in de novo UC^29–31^ (Extended Data Fig. 2a,b and Supplementary Note 2). In sum, tamoxifen does not show a direct mutagenic effect.

### TA-UC harbors fewer mutational events in *PIK3CA* and *PIK3R1*

**Extended Data Fig. 3:**
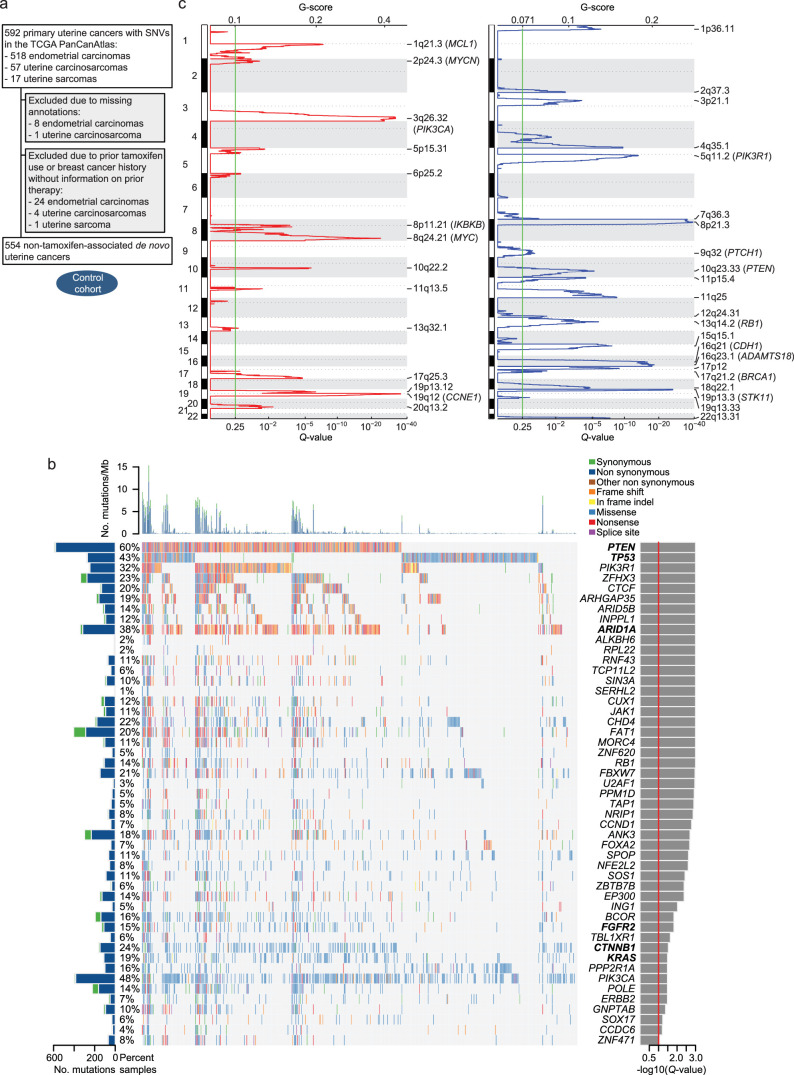
Genomic alterations in de novo UC samples from TCGA. (**a**) CONSORT flow diagram of de novo UC allocation from the TCGA PanCanAtlas. (**b**) Plot of genomic features, top panel depicts mutation frequency per megabase (Mb); bottom panel depicts significantly mutated genes detected by MutSig2CV (red line, *Q* < 0.1), ordered by significance; genes significantly mutated in the TA-UC cohort are shown in bold. (**c**) Amplifications (left, red) and deletions (right, blue) detected by GISTIC. Chromosomal positions from top to bottom; *Q-*values from left to right (green line, *Q* < 0.25). Significant peaks are annotated with chromosomal position and candidate cancer genes, where applicable.

To discover mutation-based drivers of TA-UC, we used MutSig2CV (Fig. 1b–d; *Q* < 0.1) and identified four significantly mutated genes, *PTEN*, *KRAS*, *TP53* and *ARID1A*, all of which were also observed as drivers in de novo UC^29–31^ (Extended Data Fig. 3a,b and Supplementary Table 3). To increase statistical power for finding drivers using the smaller TA-UC cohort, we further restricted our analysis to 113 known UC drivers (Supplementary Table 4)^29–31,34^ to decrease the number of hypotheses tested and found that *RNF43*, *FGFR2* and *CTNNB1* were also significantly mutated (*Q* < 0.1).

Next, to evaluate the relationship between driver gene mutation frequencies and tamoxifen exposure, we assessed the statistical power for finding differences (higher or lower) between TA-UC and de novo UC samples (Methods). Among the 49 genes identified as significantly mutated drivers in de novo UC (Extended Data Fig. 3b), we found five (*PTEN*, *PIK3CA*, *TP53*, *ARID1A* and *PIK3R1*) that were powered (Methods; Bonferroni-corrected optimal Fisher’s exact *P* < 0.05; Extended Data Fig. 2c and Supplementary Table 5). We observed a significant difference in mutation frequencies for two of these genes (Fig. 1e), both in the PI3K pathway: *PIK3CA* (encoding the PI3K catalytic subunit p110α; 14% versus 48%; *P* = 0.003, *Q* = 0.007; two-sided BH-corrected Fisher’s exact test) and *PIK3R1* (encoding the PI3K regulatory subunit p85α; 0% versus 31%; *P* = 0.0009, *Q* = 0.005). Surprisingly, both genes had lower mutation frequencies in TA-UC. Stratified Fisher’s exact tests confirmed that the lower mutation frequencies in TA-UC (*PIK3CA*, combined *P* = 0.008; *PIK3R1*, combined *P* = 0.001) were not driven by the different distributions of tumor grades in our TA-UC and de novo UC cohorts (Supplementary Note 3 and Supplementary Fig. 1a).

To search for additional genes among the 113 known UC drivers with reduced mutation frequency in TA-UC, we used a one-sided test and found 30 genes for which we had sufficient power to detect reduced mutation frequency (Methods). Again, only *PIK3CA* (*P* = 0.002, *Q* = 0.03; one-sided BH-corrected Fisher’s exact test) and *PIK3R1* (*P* = 0.0004, *Q* = 0.01) reached significance (Extended Data Fig. 2d and Supplementary Table 6).

**Extended Data Fig. 4:**
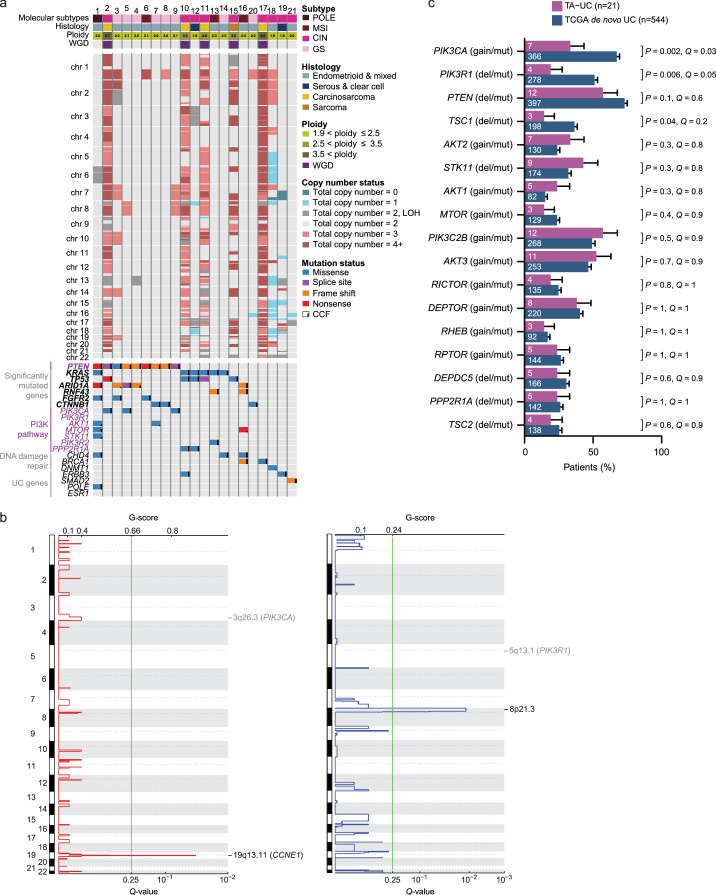
Copy number changes in TA-UC. (**a**) TA-UC samples are grouped according to mutated genes in Fig. 1b, with each column representing one tumor. *Top*: Sample identifiers of the TA-UC discovery cohort; molecular (MSI, microsatellite instability; CIN, chromosomally instability; GS, genomically stable; POLE, polymerase ε) and histological classifications; ABSOLUTE-generated ploidy values; presence of whole-genome doubling (WGD). *Middle*: ABSOLUTE total copy numbers of individual segments delineated by their genomic position along the 22 chromosomes (top to bottom); colors indicate loss, copy neutral loss-of-heterozygosity (LOH) or gain at genomic loci. *Bottom*: colored boxes indicate presence of a mutation; bars on the right of the boxes depict cancer cell fraction (CCF); significantly mutated genes are shown in bold as in Fig. 1b; genes of the PI3K pathway are in violet. (**b**) Amplifications (left, red) and deletions (right; blue) detected by GISTIC in the TA-UC discovery cohort. Chromosomal positions from top to bottom; *Q-*values from left to right (green line, *Q* < 0.25). Significant peaks are annotated with chromosomal position and candidate cancer genes, where applicable (black); positions of non-significant genes of the PI3K pathway are also annotated (gray). (**c**) Bar plot of tumors with genomic alterations in key PI3K pathway genes including single-nucleotide variants (mut) and somatic copy number alterations (gain/deletion); only TCGA tumors with both data types considered; genes altered by either type counted once per tumor; bars represent mutation frequencies, with genes ordered by *P*-value (top to bottom); error bars reflect standard deviation from the β-distribution; significance analysis by two-sided Fisher’s exact test with and without Benjamini-Hochberg procedure; numbers in bars indicate mutated tumor count per group.

Compared to de novo UC, TA-UC also had significantly fewer hotspot *PIK3CA* mutations (10% versus 38%; *P* = 0.009, Fisher’s exact test; Fig. 1f and Supplementary Table 7), which confer stronger pathway activation^35^. This observation held true even when controlling for gene coverage (Extended Data Fig. 2e and Supplementary Note 4) and was validated by droplet digital PCR (ddPCR; Extended Data Fig. 2f and Supplementary Note 5). Of note, we identified two patients in the TCGA cohort exposed to tamoxifen before UC diagnosis (Methods) who did not harbor a *PIK3CA* mutation (Fig. 1f). Finally, genomic identification of significant targets in cancer (GISTIC) analysis^36^ (Methods) did not detect significant enrichments of *PIK3CA* amplifications and *PIK3R1* deletions in TA-UC (Extended Data Fig. 4a,b) compared to de novo UC (*Q* < 0.25; Extended Data Fig. 3c), ruling out the possibility that SCNAs account for the lack of *PIK3CA* and *PIK3R1* single-nucleotide variants (SNVs) in TA-UC. Together, even when considering SNVs and SCNAs, *PIK3CA* (33% versus 67%; *P* = 0.002; Fisher’s exact test) and, to a lesser statistical extent, *PIK3R1* (19% versus 51%; *P* = 0.006) remained significantly less altered in TA-UC than in de novo UC (Fig. 1g), distinguishing these two genes, especially *PIK3CA*, from other PI3K pathway genes^37^ in TA-UC (Extended Data Fig. 4c).

**Extended Data Fig. 5:**
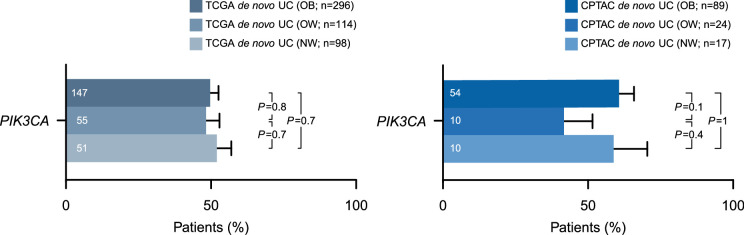
*PIK3CA* mutation frequencies in de novo UC by body mass. Bar plots of TCGA (left) and CPTAC (right) de novo UC cohorts; bars represent *PIK3CA* mutation frequencies across three body mass index (BMI) groups: normal weight (NW, BMI < 25), overweight (OW, BMI 25 – 29.9), and obese (OB, BMI ≥ 30). Error bars reflect standard deviation from the β-distribution; significance analysis by two-sided Fisher’s exact test; numbers in bars indicate mutated tumor count per group.

We further investigated whether obesity, a surrogate for higher estrogen^38–40^ due to its association with elevated endogenous estrogen levels^41^, a known UC risk factor^42^, has effects similar to tamoxifen. Of note, obesity is not a surrogate for exogenous unopposed estrogen exposure as in hormone replacement treatment, which is associated with a higher UC risk^43,44^. We found no significant differences in *PIK3CA* mutation frequencies across obesity categories (all *P* ≥ 0.1; Extended Data Fig. 5 and Supplementary Note 6). To more directly assess the differential effects of estrogen and tamoxifen, we performed transcriptomic analysis of human endometrial cells, which showed upregulation of PI3K pathway genes after tamoxifen, but not estradiol (E2), treatment (Supplementary Fig. 2a,b and Supplementary Note 7). These findings suggest that tamoxifen activates the PI3K pathway, which is commonly mutationally activated in de novo UC, and provide evidence that tamoxifen and E2 have different effects on the uterus.

### Cohorts validate low *PIK3CA* mutation frequency in TA-UC

**Fig. 2:**
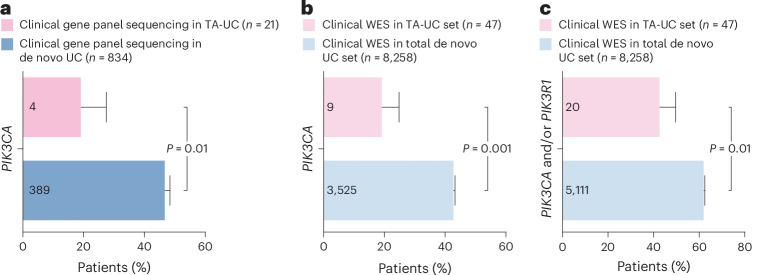
Independent clinical TA-UC cohorts confirm reduced *PIK3CA* mutation frequency. **a**, Bar plot of clinical gene panel sequencing for TA-UC and de novo UC; bars represent *PIK3CA* mutation frequencies; error bars reflect s.d. from the β-distribution; significance analysis by two-sided Fisher’s exact test; numbers in bars indicate mutated tumor count per group. **b**, Bar plot of clinical WES data for TA-UC and de novo UC; bars represent *PIK3CA* mutation frequencies; error bars reflect s.d. from the β-distribution; numbers in bars indicate mutated tumor count per group. Significance analysis by two-sided Fisher’s exact test. **c**, Bar plot of WES data for TA-UC and de novo UC; bars represent *PIK3CA* and/or *PIK3R1* mutation frequencies; error bars reflect s.d. from the β-distribution; numbers in bars indicate mutated tumor count per group. Significance analysis by two-sided Fisher’s exact test.

**Extended Data Fig. 6:**
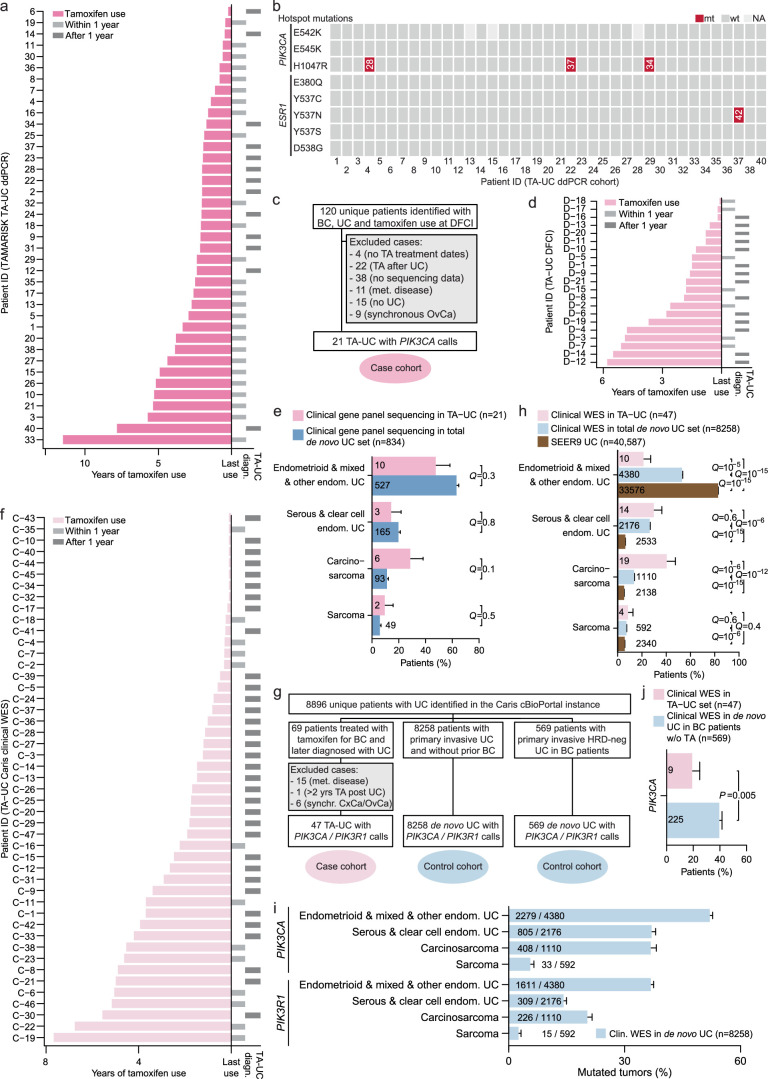
TA-UC validation cohorts. (**a**, **d**, **f**) Time course plots for patients in the TAMARISK validation cohort (**a**), the clinical gene panel sequencing cohort (**d**), and the clinical whole-exome sequencing (WES) validation cohort (**f**), showing duration of tamoxifen (TA) treatment (colored bars) and periods of uterine cancer (UC) diagnosis (diagn., gray bars). (**b**) Integrated plot of *PIK3CA* and *ESR1* hotspot mutations in TA-UC (TAMARISK validation cohort), detected by droplet digital PCR (ddPCR; red; variant allelic fraction [VAF, %] shown); each column represents one tumor (NA, not available). (**c**, **g**) CONSORT flow diagrams showing patient allocation (BC, breast cancer; CxCa, cervical cancer; HRD, homologous recombination deficiency; met, metastatic; OvCa, ovarian cancer; synchr, synchronous; UC, uterine cancer; yrs, years) for clinical gene panel sequencing at Dana-Farber Cancer Institute (DFCI; **c**) and clinical whole-exome sequencing (WES; **g**). (**e**, **h**) Bar plots showing frequencies of histological uterine cancer (UC) types (endom, endometrial) in patients with and without history of tamoxifen (TA) from clinical gene panel sequencing (**e**) and clinical whole-exome sequencing (WES) compared to SEER9 data (**h**); error bars reflect the standard deviation from the β-distribution; numbers in/above bars indicate tumor count per group; significance analysis by two-sided Fisher’s exact test with Monte Carlo Benjamini-Hochberg procedure. (**i**) Bar plot of clinical (clin) whole-exome sequencing (WES) data from patients with de novo UC (endom, endometrial); bars show *PIK3CA* and *PIK3R1* mutation frequencies, grouped by histological subtype; numbers in/above bars indicate number of mutated samples (before the slash) and total number of samples in that subtype (after the slash); error bars reflect the standard deviation from the β-distributions. (**j**) Bar plot of clinical WES data from breast cancer patients with and without history of tamoxifen (TA); bars show mutation frequencies; error bars reflect the standard deviation from the β-distribution; numbers in bars indicate mutated tumor count per group. Significance analysis by two-sided Fisher’s exact test.

In our validation analysis, we prioritized *PIK3CA* for two reasons: (1) in UC, *PIK3CA* is more frequently mutated than *PIK3R1* (Extended Data Fig. 3b), allowing for a more statistically powerful analysis and (2) unlike *PIK3R1*, which may require additional factors for PI3K pathway regulation, *PIK3CA* directly activates this pathway, making results more interpretable. We confirmed our results from our discovery cohort in three validation cohorts. First, we analyzed an additional 39 TA-UCs from the TAMARISK study (Supplementary Table 1 and Extended Data Fig. 6a) for *PIK3CA* hotspot mutations (E542K, E545K, H1047R) and detected three (8%) by ddPCR (Extended Data Fig. 6b), which is lower but consistent with the 14% ddPCR-defined hotspots in our discovery cohort (Extended Data Fig. 2f). Second, a clinical database cohort subjected to gene panel sequencing (Extended Data Fig. 6c–e and Supplementary Tables 1, 8 and 9) confirmed the low *PIK3CA* mutation frequency in TA-UC (19% versus 47%; *P* = 0.01; Fig. 2a). This was not attributable to differences in population descriptors between TA-UC and de novo UC (combined *P* = 0.02; stratified Fisher’s exact test; Supplementary Note 3 and Supplementary Fig. 1b). Third, analysis of another clinicogenomic dataset (Extended Data Fig. 6f,g and Supplementary Tables 1 and 10) corroborated a lower *PIK3CA* mutation frequency in TA-UC (19%) compared to de novo UC (43%; *P* = 0.001; Fig. 2b). However, histological subtype frequencies in this dataset differed from the general patient population (based on SEER9 data) and further varied between TA-UC and de novo UC (Extended Data Fig. 6h and Supplementary Table 9). To address this potential confounding factor, we performed a stratified Fisher’s exact test, which confirmed the lower *PIK3CA* mutation frequency in TA-UC (combined *P* = 0.01). Building on this, we next explored subtype-specific differences and extended our analysis to include both *PIK3CA* and *PIK3R1* mutation frequencies. Given the smaller sample sizes in some subtypes, we first calculated the statistical power to detect differences in mutation frequency between groups (Methods). Of the three powered subtypes (Bonferroni-corrected (*n* = 8) optimal *P* value < 0.05), endometrioid, mixed and other, and serous and clear cell endometrial UC showed significantly lower *PIK3CA* mutation frequencies (20% versus 52%, *P* = 0.04; 7% versus 37%, *P* = 0.01; one-sided Fisher’s exact test; Supplementary Table 11). However, the dataset was underpowered to detect differences in *PIK3R1* mutation frequencies between TA-UC and de novo UC. This is consistent with the generally lower frequency of *PIK3R1* mutations than *PIK3CA* mutations in de novo UC (26% versus 43%; *P* < 2 × 10^−16^; two-sided Fisher’s exact test; Extended Data Fig. 6i), suggesting that larger datasets are needed to test for differences in *PIK3R1* mutations. However, to address this with the existing data, as *PIK3CA* and *PIK3R1* together encode the enzyme PI3K, we analyzed the combined mutation status and found that *PIK3CA*- and/or *PIK3R1-*mutated tumors were less frequent in TA-UC (*P* = 0.01; Fig. 2c). Thus, this is consistent with our hypothesis that PI3K signaling represents a molecularly distinct feature of TA-UCs.

We took a conservative approach by including only de novo UC from patients without a history of breast cancer as controls to confidently exclude patients with potential undocumented tamoxifen treatment. However, to further isolate the effect of tamoxifen on *PIK3CA* mutation frequencies, we also compared clinicogenomic TA-UCs with a unique cohort of de novo UC from patients with breast cancer never treated with tamoxifen. Here, TA-UC also had a significantly lower *PIK3CA* mutation frequency (*P* = 0.005; two-sided Fisher’s exact test; Extended Data Fig. 6j). Thus, a history of a breast cancer diagnosis before UC diagnosis cannot explain the lower frequency of *PIK3CA* mutations observed in TA-UC compared to de novo UC. Collectively, the consistent finding of a lower frequency of *PIK3CA* mutations in TA-UC across multiple cohorts, including real-world cohorts, supports a tamoxifen-specific effect and highlights the relevance of this discovery to clinical practice.

Most TA-UCs (12 of 21) and de novo UCs (472 of 554) in the discovery cohorts had at least one SNV event in a PI3K pathway gene^37^ (Extended Data Fig. 4c). Consistent with previous reports^45^, multiple PI3K-related genes were often mutated within individual samples in both cohorts (Extended Data Fig. 2g). However, TA-UC had a lower number of concurrent PI3K pathway mutations (median of one event per sample, range of 0–6) than de novo UC (median of two events per sample, range of 0–45; *P* = 0.0002), suggesting fewer potential driver events that activate PI3K signaling in TA-UC. We explored the oncogenic role of *PIK3CA* mutations in the context of other PI3K pathway events and observed a significant co-occurrence of *PTEN* mutations with *PIK3CA* mutations in de novo UC (odds ratio = 2, *P* = 0.007; Fisher’s exact test), reflecting their known complementary but distinct functional roles^29,46^. By contrast, this co-occurrence was not observed in TA-UC (*P* = 0.07), despite a similar frequency of *PTEN* mutations (*Q* = 0.2, BH-corrected Fisher’s exact test; Fig. 1e). In addition, we observed almost complete mutual exclusivity between tamoxifen use (using our discovery cohorts and two TCGA patients with TA-UC) and *PIK3CA* mutations (odds ratio = 0.2, *P* = 0.001; Fisher’s exact test). In aggregate, these observations support the hypothesis that tamoxifen may act as an alternative mechanism for PI3K pathway activation in the absence of *PIK3CA* mutations.

### In vivo studies support tamoxifen-induced PI3K signaling

**Fig. 3:**
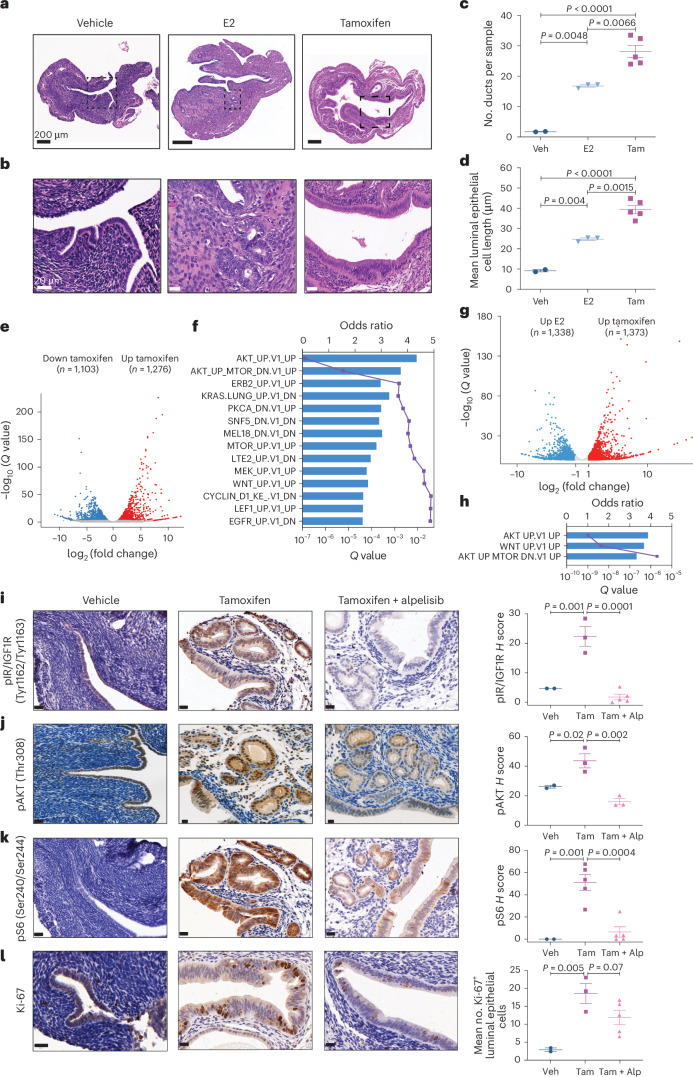
Tamoxifen affects cell morphology and PI3K signaling in mouse endometrial epithelial cells. **a**,**b**, Representative hematoxylin and eosin (H&E)-stained endometrial sections from oophorectomized mice treated with vehicle, E2 or tamoxifen. Scale bars, 200 μm in **a**, 20 μm in **b**. **c**,**d**, Quantification of endometrial changes with number of ducts per mouse in **c** and mean length of luminal epithelial cells in **d**. Each symbol represents the mean of six sections per biologically independent mouse; sample sizes: vehicle (Veh), *n* = 2 (small horn size and extensive fibrosis in the region surrounding the horns secondary to the oophorectomy made dissection difficult); E2, *n* = 3; tamoxifen (Tam), *n* = 5. Center line depicts median; error bars represent s.e.m.; significance analysis using one-way ANOVA. **e**, Volcano plot depicts differentially expressed genes identified using DESeq2 by comparing tamoxifen versus vehicle in endometrial epithelial cells (*Q* < 0.01, BH-corrected two-sided Wald test). Red indicates upregulated (log_2_ (FC) > 1), blue indicates downregulated (log_2_ (FC) < −1), and genes not significantly changed are gray. **f**, Pathway enrichment analysis on the differently expressed genes from **e**. Bar plot depicts the odds ratio of pathway enrichment of MSigDB oncogenic signatures (gene set names shown on the *y* axis) in tamoxifen-versus-vehicle upregulated genes (log_2_ (FC) > 2, *Q* < 0.01, DESeq2); purple line indicates *Q* values from BH-corrected two-sided Fisher’s exact tests. **g**, Differentially expressed genes comparing tamoxifen versus E2 treatment, analyzed as described in **e**. **h**, Pathway enrichment analysis of the differently expressed genes from **g**. Bar plot depicts the odds ratio of pathway enrichment in tamoxifen-upregulated genes when compared to E2 treatment, analyzed as described in **f**. **i**–**l**, Left: representative immunohistochemistry (IHC) images with H&E counterstaining showing expression (brown) of phospho-insulin receptor (pIR) or IGF1R (Tyr1162/Tyr1163) in **i**, phospho-AKT (pAKT) (Thr308) in **j**, pS6 (Ser240/Ser244) in **k** and Ki-67 in **l** in the endometrial epithelium from mice treated with vehicle, tamoxifen or tamoxifen plus alpelisib (Tam + Alp). Scale bars, 20 μm. Right: quantification of immunoreactivity shown as *H* scores (product of percent positive cells × signal intensity in optical density). Each symbol represents the mean of five regions per biologically independent mouse, imaged at 20× magnification; sample sizes: vehicle, *n* = 2 (small horn size and surrounding fibrosis secondary to oophorectomy made dissection difficult); tamoxifen (**i**,**j**,**l**, *n* = 3; **k**, *n* = 5); tamoxifen and alpelisib (**i**,**k**,**l**, *n* = 5; **j**, *n* = 3). Center line depicts median; error bars represent s.e.m. Significance analysis by one-way ANOVA.

To test the hypothesis that tamoxifen-mediated activation of ER affects PI3K signaling in the uterus, we performed in vivo studies in mice, initially analyzing the effects of E2 and tamoxifen on ER in the uterus. Because most UCs, including TA-UCs, develop in postmenopausal women^47^, we performed these experiments under postmenopausal conditions. To test the effects of E2, we used a relatively low dose to reflect the lower, clinically acceptable doses of exogenous estrogen currently permitted due to the risk of UC with unopposed estrogen^43,44^. Female C57BL/6 mice were oophorectomized after sexual maturity and treated with (1) vehicle control (E2 deprived), (2) E2 or (3) tamoxifen, and uteri were collected 30 d after treatment. The uteri from the vehicle control showed an atrophic epithelial lining composed of a single layer of flattened cells devoid of glands (Fig. 3a,b), confirming E2 dependency of endometrial epithelial cells. As expected, E2 supplementation promoted duct proliferation (mean number of ducts per mouse in E2 (16.8) versus vehicle (1.7), *P* = 0.0048; one-way ANOVA with Tukey correction; Fig. 3c) and enhanced cell growth (mean length of luminal epithelial cells per mouse in E2 (24.7 µm) versus vehicle (9.2 µm), *P* = 0.004; Fig. 3d). Tamoxifen enhanced the increase in the number of ducts and cell length compared to E2 (mean number of ducts per mouse in tamoxifen (28.1) versus E2 (16.8), *P* = 0.007; mean length of luminal epithelial cells per mouse in tamoxifen (39.4 µm) versus E2 (24.7 µm), *P* = 0.0015; Fig. 3c,d), suggesting that the effects of tamoxifen on the endometrium are distinct from those of E2 at these doses.

**Extended Data Fig. 7:**
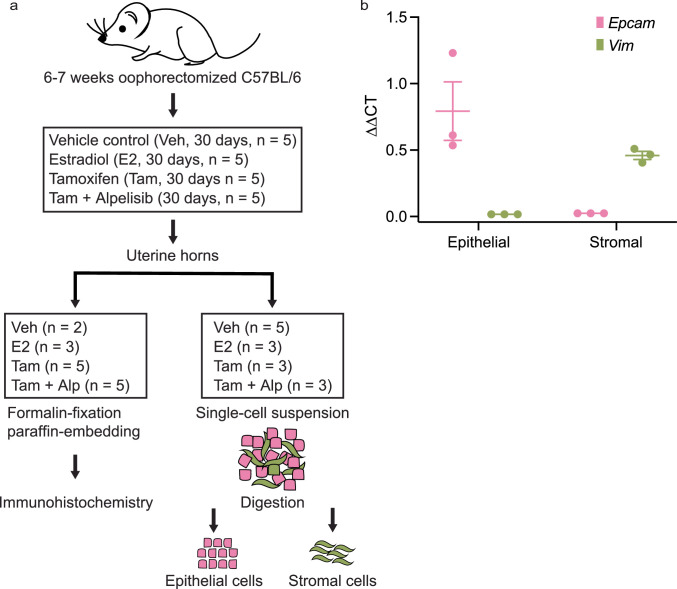
Mouse endometrial epithelial cell purification. (**a**) Schematic illustrating the experimental design for the collection of uterine horns, isolation of endometrial epithelial cells and downstream analyses. Uterine horns were used for formalin fixation and paraffin embedding followed by immunohistochemistry (left) and for RNA sequencing (RNA-seq, right) from mice as indicated. (**b**) Relative quantitative reverse transcription PCR (qRT-PCR) of *Epcam* and *Vim* mRNA expression in mouse endometrial cell populations isolated from a tamoxifen-treated mouse (n = 1) to confirm the purity of the isolated cell fractions. *Gapdh* was used as reference. Each symbol represents a technical replicate; center line depicts mean; error bars represent SEM.

**Extended Data Fig. 8:**
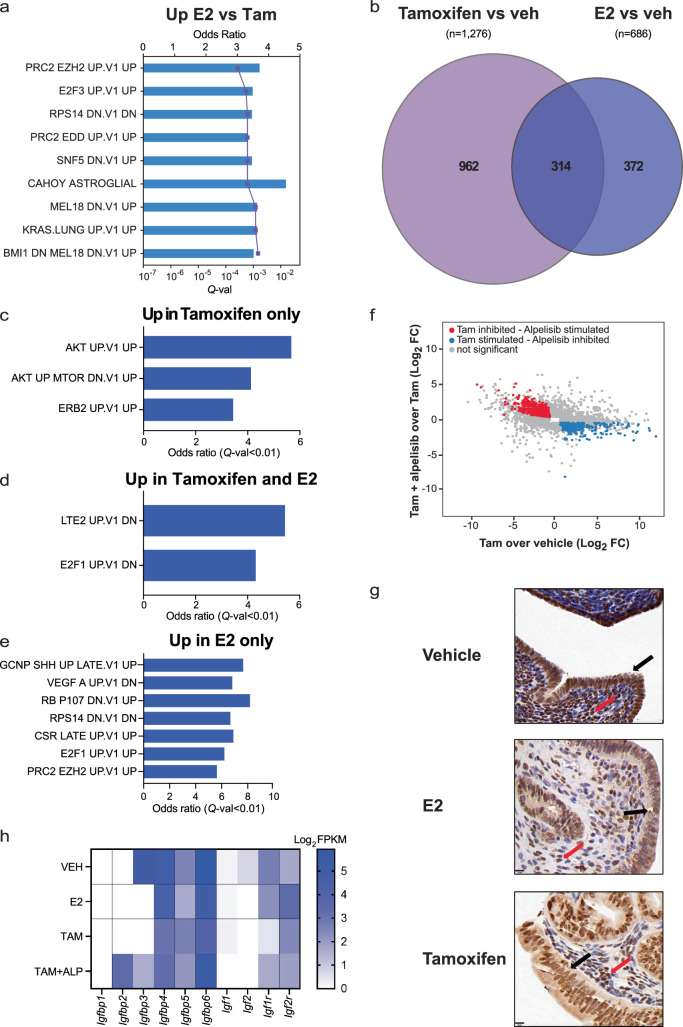
Effects of tamoxifen and E2 in mouse endometrial epithelial cells. (**a**) Pathway enrichment analysis on the differently expressed genes identified by DEseq2 from comparing estradiol (E2) versus tamoxifen (Tam) in endometrial epithelial cells (*Q* < 0.01, Benjamini-Hochberg-corrected two-sided Wald test). Bar plot depicts the odds ratio of pathway enrichment (MSigDB oncogenic signatures); purple line indicates the *Q*-values from the Benjamini-Hochberg-corrected two-sided Fisher’s exact tests. (**b**) Venn diagram showing the genes upregulated after tamoxifen treatment compared to vehicle (veh) control, and genes upregulated after E2 compared to veh control (DEseq2, log_2_FC > 1, *Q* < 0.01, Benjamini-Hochberg-corrected two-sided Wald test). (**c**–**e**) Pathway enrichment analysis (MSigDB oncogenic signatures) on: (**c**) genes upregulated with tamoxifen treatment versus vehicle that are not shared with the E2-upregulated genes (n = 962, as shown in the Venn diagram in **b**); (**d**) shared genes upregulated by tamoxifen treatment versus vehicle and E2 treatment versus vehicle; and (**e**) genes upregulated by E2 treatment versus vehicle but not upregulated by tamoxifen versus vehicle. Bar plots depict the odds ratio of pathway enrichment, *Q*-values from the Benjamini-Hochberg-corrected two-sided Fisher’s exact tests. (**f**) Comparison of differentially expressed genes (log_2_ fold change [FC]) between tamoxifen (tam) over vehicle control (x-axis) and tamoxifen plus alpelisib (y-axis). Genes with FDR < 0.05 (DEseq2) are categorized and color-coded. (**g**) Representative immunohistochemistry images of ER expression (nuclear brown staining) in epithelial (black arrow) and stromal cells (red arrow) in the uteri from mice treated with vehicle control, estrogen (E2), and tamoxifen. Scale bars: 10μm, H&E counterstaining. Each treatment group represents independently repeated experiments with similar results: vehicle (n = 2 mice), E2 (n = 3 mice), and tamoxifen (n = 5 mice). (**h**) Heatmap of log_2_ FPKM expression levels (scale 0-5) of genes related to *Igf1r* in mouse endometrial epithelial cells from vehicle (Veh), estradiol (E2), tamoxifen (Tam) and tamoxifen plus alpelisib (Tam+Alp) treatment cohorts.

To identify how tamoxifen increases epithelial cell proliferation through ER and, more specifically, to test the role of the PI3K pathway, we performed differential gene expression analysis of RNA sequencing (RNA-seq) from single-cell suspensions of endometrial epithelial cells isolated from mice treated with vehicle control, E2, tamoxifen or tamoxifen plus alpelisib, an α-selective PI3K inhibitor^48^ (Extended Data Fig. 7a,b). DESeq2 analysis identified 1,276 upregulated (log_2_ (fold change (FC)) > 1; *Q* < 0.01, BH-corrected Wald test) and 1,103 downregulated (log_2_ (FC) < −1; *Q* < 0.01) genes in the tamoxifen- versus vehicle-treated mice (Fig. 3e and Supplementary Table 12). Pathway analysis of genes upregulated after tamoxifen treatment showed enrichment in genes involved in the receptor tyrosine kinase (RTK)–PI3K–AKT signaling pathway (Fig. 3f). As most de novo UCs express ER and are associated with ER activation^49^, we assessed differences between tamoxifen and E2 treatment. We identified 1,373 upregulated and 1,338 downregulated genes in tamoxifen- versus E2-treated endometrial epithelial cells, respectively (|log_2_ (FC)| > 1; *Q* < 0.01; Fig. 3g). Genes upregulated after tamoxifen treatment were enriched in genes involved in the PI3K–AKT–mechanistic target of rapamycin (mTOR) and WNT signaling pathways (Fig. 3h). By contrast, genes upregulated with E2 supplementation were enriched in gene sets associated with enhancer of zeste 2 polycomb repressive complex 2 subunit (EZH2) knockdown (PRC2 EZH2 UP.V1 UP) and proliferation (E2F3 UP.V1 UP; Extended Data Fig. 8a). Furthermore, when comparing tamoxifen or E2 to vehicle, 314 tamoxifen-upregulated genes (of the 1,276 in Fig. 3e) overlapped with the E2-upregulated genes (*n* = 686, log_2_ (FC) > 1; *Q* < 0.01 versus vehicle; Extended Data Fig. 8b). Pathway analysis showed that genes uniquely upregulated by tamoxifen but not genes upregulated by E2 alone or by both tamoxifen and E2 were enriched in the AKT–mTOR pathway (Extended Data Fig. 8c–e). Thus, the effects of tamoxifen over 30 d were distinct from those of E2 at this dose in terms of the AKT–mTOR pathway. Lastly, the addition of alpelisib to tamoxifen significantly downregulated tamoxifen-upregulated genes (Extended Data Fig. 8f and Supplementary Table 13), indicating that the effect of tamoxifen was at least partially through PI3K signaling.

We next deciphered key components of the tamoxifen–PI3K signaling axis. Crosstalk between ER and the PI3K–AKT pathway is well described^50,51^. ER mediates insulin-like growth factor 1 (IGF1) synthesis, which activates the IGF1 receptor (IGF1R), followed by downstream PI3K–AKT pathway activation. IGF1-stimulated IGF1R can also activate ER, at least in part through PI3K–AKT-mediated phosphorylation of ER, creating a positive feedback loop^52,53^. We therefore interrogated the impact of tamoxifen and alpelisib treatment on the IGF1R–PI3K–AKT axis in the uterus. Indeed, tamoxifen-activated IGF1R–PI3K–AKT signaling was evidenced by the significant increase in phospho-IGF1R (*P* = 0.001; one-way ANOVA; Fig. 3i), phospho-AKT (*P* = 0.02; Fig. 3j) and phospho-S6 (*P* = 0.001; Fig. 3k). Alpelisib abrogated the tamoxifen-induced increase in PI3K–AKT signaling, IGF1R activation (Fig. 3i–k) and cell proliferation (Fig. 3l), suggesting that tamoxifen-induced proliferation occurs via ER and IGF1R crosstalk-mediated activation of PI3K signaling.

**Fig. 4:**
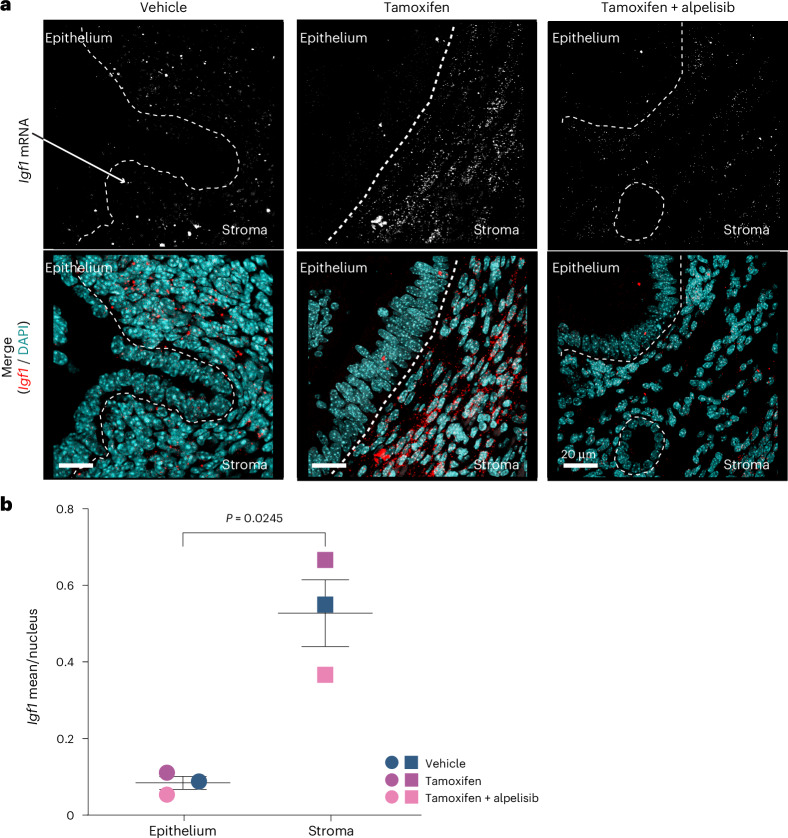
*Igf1* expression in mouse endometrial stromal cells. **a**, Representative RNAscope images of *Igf1* expression in mouse uteri (three mice, treated as indicated). Dashed white lines depict the border between the epithelium and the stroma. White foci represent *Igf1* mRNA signal (top); merged images (bottom) show 4′,6-diamidino-2-phenylindole (DAPI) (teal) and *Igf1* (red). Different contrast settings were used for top and bottom images of the vehicle control. Scale bars, 20 μm. **b**, Mean *Igf1* staining intensity per nucleus across entire uterine tissue areas (epithelium and stroma) per biologically independent mouse (*n* = 3). Significance analysis by paired two-sided *t*-test. Center line depicts median; error bars represent s.e.m.

Because ER is expressed in both endometrial epithelial and stromal cells independent of treatment conditions (Extended Data Fig. 8g), and previous studies provided conflicting data for a paracrine versus autocrine effect^54,55^, we next asked how the tamoxifen-mediated effect on ER activates the IGF1R–PI3K–AKT pathway in the uterus. We examined the transcriptomic levels of *Igf1* and *Igf2* as well as their receptors (*Igf1r*, *Igf2r*) and IGF-binding proteins (*Igfbp1*–*Igfbp6*) in endometrial epithelial cells in uteri from mice treated with vehicle control, E2 and tamoxifen with or without alpelisib. Tamoxifen-treated mice showed a significant decrease in *Igfbp3*, *Igfbp4* and *Igfbp6* transcript levels compared to vehicle control (*Igfbp3*, log_2_ (FC) = −7, *Q* = 6 × 10^−37^, DESeq2; *Igfbp4*, log_2_ (FC) = −1.7, *Q* = 2 × 10^−5^; *Igfbp6*, log_2_ (FC) = −1.7, *Q* = 3 × 10^−5^; Extended Data Fig. 8h). As IGF-binding proteins, particularly IGFBP3, regulate the bioavailability of IGF in circulation and in the cell^56^, these decreased levels suggest a possible cell-intrinsic tamoxifen-mediated effect by which IGF1 has increased availability upstream of PI3K–AKT in endometrial epithelial cells. The addition of the PI3K inhibitor alpelisib to tamoxifen increased *Igfbp3* (log_2_ (FC) = 4, *Q* = 1.5 × 10^−12^) and *Igfbp6* (log_2_ (FC) = 1.7, *Q* = 6.2 × 10^−13^) levels (Extended Data Fig. 8h). Given the low *Igf1* messenger RNA (mRNA) levels observed in mouse epithelial endometrial cells in all four conditions in the RNA-seq data (Extended Data Fig. 8h), we used RNAscope, an in situ hybridization assay, to detect mRNA within the intact tissue architecture. Consistent with the RNA-seq data, *Igf1* levels were low in endometrial epithelial cells and predominantly detected in the stroma (*P* = 0.025, paired two-sided *t*-test; Fig. 4a,b). These results suggest that tamoxifen-induced activation of the IGF1R–PI3K axis in endometrial epithelial cells is potentially mediated by paracrine (IGF1 secreted by stromal cells) and cell-intrinsic (decreased levels of IGFBP3 in epithelial cells) effects. Together, our in vivo and genomic findings suggest that tamoxifen activates PI3K signaling, contributing to increased cell proliferation and likely uterine carcinogenesis independent of oncogenic *PIK3CA* mutations.

### TA-UCs have fewer clonal driver mutations

Our preclinical findings showed that PI3K pathway activation by tamoxifen occurs in a short period of time. We therefore sought to understand the timing of driver events in TA-UC and infer the early events in TA-UC compared to de novo UC and clonally expanded normal endometrial cells.

**Fig. 5:**
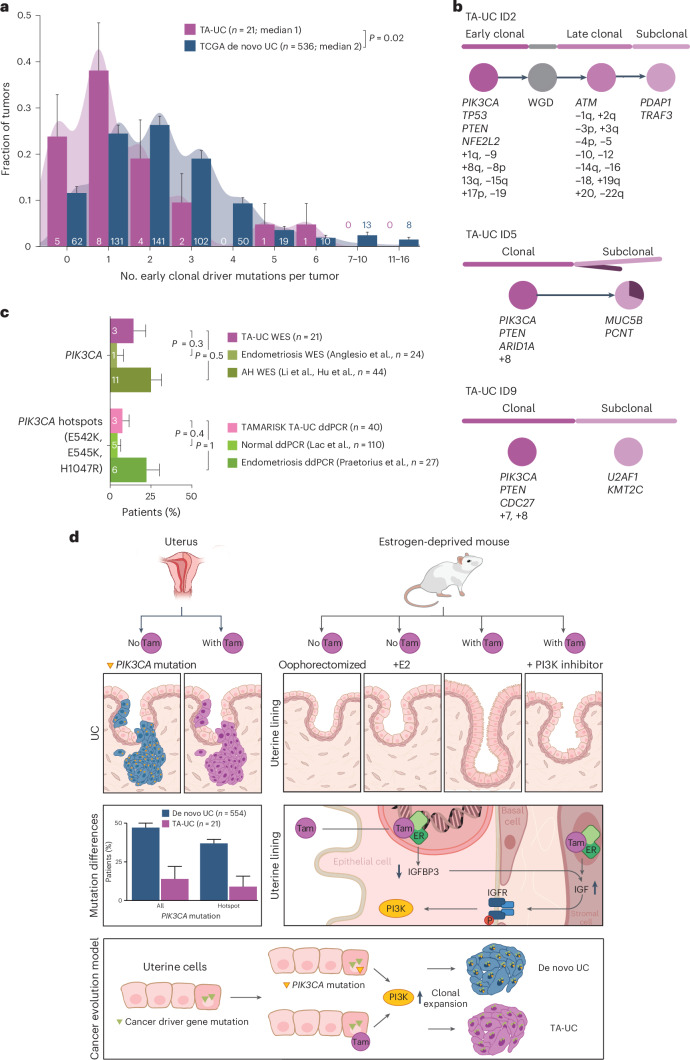
Mutations in *PIK3CA* are early events in tumorigenesis. **a**, Density histogram with bars representing fraction of tumors grouped by number of clonal mutations in commonly mutated early driver genes (Supplementary Table 14) per sample; error bars reflect s.d. from the β-distribution; significance analysis by two-sided Wilcoxon test; numbers in or above bars indicate the mutated tumor count per group. **b**, Estimated phylogenetic trees (top), relative order and molecular timing of events (bottom) in *PIK3CA-*mutated TA-UC (discovery cohort). Circle plots indicate estimated clonal composition. **c**, Bar plot of WES and ddPCR data for TAMARISK TA-UC samples, normal endometrial tissue^62^ and benign endometrial disease endometriosis^63,64^ and atypical hyperplasia^65,66^ (AH); bars represent *PIK3CA* mutation frequencies; error bars reflect s.d. from the β-distribution; numbers in bars indicate mutated tumor count per group; significance analysis by two-sided Fisher’s exact test. **d**, Schematic illustration depicting (1) *PIK3CA* mutations in TA-UC and de novo UC (top two left subpanels; bars represent mutation frequencies; error bars reflect s.d. from the β-distribution), (2) the in vivo mouse model (top right) with cell morphological changes from normal atrophic (no tamoxifen (Tam)) and normal proliferative (+E2) to increased number of ducts and cell hypertrophy (with tamoxifen) and normalized number of ducts and cell length (with tamoxifen and PI3K inhibitor), (3) the model of PI3K signaling induced by tamoxifen (middle right) and (4) the model of UC evolution for de novo UC and TA-UC (bottom).

First, using discovery WES data and our PhylogicNDT suite of tools^57,58^, we identified early clonal driver mutations in TA-UC and de novo UC (Supplementary Table 14). Comparing these events between cohorts, we found no difference in the timing of early driver events (Extended Data Fig. 2h). However, TA-UC harbored significantly fewer early genomic events per sample (median, one event) than de novo UC (median, two events; *P* = 0.02; Wilcoxon test; Fig. 5a). The shift was not significantly larger than one event (TA-UC events + 1 versus de novo UCs, *P* = 0.4), leading us to hypothesize that tamoxifen-associated perturbation of the PI3K signaling pathway acts as the missing driver event toward malignant transformation in the uterus.

We next analyzed the timing of *PIK3CA* mutations in TA-UC, focusing on the small subset of patients in whom *PIK3CA* mutations were detected. Although the overall number of *PIK3CA* mutations in TA-UC was lower than expected, we identified three patients with *PIK3CA* mutation by WES and one additional patient with *PIK3CA* mutation by ddPCR (Supplementary Note 5) in our discovery cohort. One possible explanation for this finding could be shorter tamoxifen exposure. However, no significant difference in intake time was observed between these four patients and the other ones with TA-UC (mean, 4.4 versus 3.6 years in mutant versus others; *P* = 0.4). A second, alternative explanation is that these cases occurred by chance. Given previous calculations of a fivefold increase in absolute UC risk due to tamoxifen (from 0.5% in women not treated with tamoxifen to ~2.5% in women receiving tamoxifen over 10 years)^59^, we expect approximately four women of our 21 patients with TA-UC to develop UC unrelated to tamoxifen treatment. This is consistent with the observed frequency of four *PIK3CA* mutations. Of note, all three *PIK3CA* mutations detected by WES, for which we could experimentally determine the cancer cell fraction (CCF), were clonal (CCF = 1; Extended Data Fig. 4a). More specifically, these mutations were often early events, preceding whole-genome duplication (WGD; Fig. 5b). Together, these findings are consistent with the presence of *PIK3CA* mutations at early stages of cancer development and align with previous observations that the mutational activation of *PIK3CA* is an early oncogenic event in UC^60^. Given that clonally expanded normal endometrial cells can also harbor *PIK3CA* mutations^61^, PI3K signaling activation might have occurred before UC initiation (and tamoxifen treatment). To test this, we compared *PIK3CA* mutation frequencies between TA-UC and three noncancerous tissue types: untreated normal endometrium^62^, benign disease endometriosis^63,64^ and atypical hyperplasia^65,66^. TA-UC and noncancerous tissue had similar *PIK3CA* mutation frequencies, a finding supported by both the TAMARISK discovery and validation cohorts (all *P* > 0.2, Fisher’s exact test; Fig. 5c). In aggregate, our observations that *PIK3CA* mutations typically occur early in tumorigenesis or even before cancer onset highlight the importance of PI3K signaling as a driver event in UC in general. Their presence in TA-UC suggests that not all UCs in patients receiving tamoxifen are driven by tamoxifen-induced PI3K signaling. While tamoxifen likely mimics the role of *PIK3CA* mutations, it does not prevent tumors from acquiring these mutations independently. However, tamoxifen decreases the selective advantage of these mutations, thereby reducing their frequency in TA-UC (Fig. 5d).

## Discussion

In summary, we describe a previously uncharacterized mechanism of oncogenesis that promotes therapy-associated secondary cancer. In addition to the known mechanisms, including treatment-associated mutagenesis and clonal selection, we propose a nonmutagenic mechanism by which a drug activates an oncogenic pathway that is otherwise activated by driver mutations in de novo tumors.

While we found no evidence of tamoxifen being mutagenic in endometrial tissue, its effect on PI3K signaling through crosstalk with ER may eliminate the need for an additional oncogenic hit, accelerating the onset of UC and explaining the associated increased risk in tamoxifen-treated patients. The finding that tamoxifen likely confers a growth advantage to cells primed with preexisting UC driver mutations is supported by clinical observations of a higher TA-UC risk in postmenopausal women^47^ older than 65 (ref. ^20^), as mutations accumulate in normal cells with age^67^. Furthermore, the role of tamoxifen as a potential driver of PI3K signaling activation is consistent with the observation that the excess risk of UC in tamoxifen-treated patients is mainly confined to the years of active treatment^19^ and provides further reassurance to women who have completed tamoxifen treatment.

Although our discovery cohort was relatively small due to the rarity of this disease, our results of low *PIK3CA* mutation frequencies in TA-UC were validated in three independent cohorts, including real-world clinicogenomic data, and supported by in vivo evidence that tamoxifen activates PI3K signaling in the uterus. We were unable to validate our *PIK3R1* findings, which represents a limitation of the study. This is likely due to the lower overall *PIK3R1* mutation frequency^37^, indicating the need for larger datasets. Additionally, unlike our population-based discovery cohort, the validation datasets were derived from clinical databases, which may introduce bias from clinicians prioritizing sequencing of higher-risk disease, making direct validation of low-frequency mutations challenging. An alternative explanation is that *PIK3R1*, encoding the regulatory subunit p85α, may not directly drive tumorigenesis like *PIK3CA*, which encodes the catalytic subunit p110α. While *PIK3CA* mutations result in constitutive PI3K pathway activation, *PIK3R1* mutations may require additional genomic alterations to have an oncogenic effect, which we could not assess due to the lack of such data.

Consistent with previous reports demonstrating crosstalk between ER and the IGF1R–PI3K pathway^50–53^, we provide in vivo evidence that tamoxifen-induced ER activation stimulates PI3K signaling in the uterus, a response not seen with low-dose E2 supplementation. Our work also implies that this effect of tamoxifen involves an interaction between epithelial and stromal cells, ultimately instigating increased proliferation. Future studies will need to evaluate whether additional mechanisms, including those unrelated to genomic alterations, contribute to TA-UC development.

Our findings that alpelisib-mediated PI3K inhibition suppresses uterine cell proliferation suggest a strategy to prevent tamoxifen-induced UC while also supporting breast cancer treatment. In line with this, metformin, a drug known to reduce PI3K signaling^68^, was shown to inhibit tamoxifen-induced endometrial proliferation in a randomized trial^69^. Furthermore, nonmutant-selective PI3K inhibitors^48^ could potentially be exploited as a future therapeutic approach to prevent TA-UC development in patients who, in addition to tamoxifen, have multiple risk factors for UC development.

## Methods

### Ethics statement

This study complies with all relevant ethical regulations. TAMARISK specimens were obtained and sequenced with the approval of the institutional review boards (IRBs) of the Netherlands Cancer Institute (protocol CFMPB294) and the Dana-Farber Cancer Institute (DFCI) (protocol 12-049B). Approval to access clinical data from the DFCI was granted under protocols 17-000 and 11-104. All participants from both the TAMARISK and DFCI cohorts provided written informed consent, allowing their genomic and clinical data to be obtained and analyzed here. In accordance with the US Code of Federal Regulations, Title 45, Part 46, Section 104(d) (45 CFR §46.104(d)), the retrospective analysis of de-identified clinical data from Caris Life Sciences was deemed exempt by the IRB, which is the WIRB-Copernicus Group IRB (formerly known as WIRB). This exemption was granted because the data were fully de-identified and the research involved no intervention or interaction with human participants; therefore, informed patient consent was not required.

### Tamoxifen-associated uterine cancer from the TAMARISK study

We analyzed 60 primary TA-UCs from the TAMARISK study^28^, diagnosed between 1983 and 2002, for which sufficient residual tissue for DNA extraction was available (Extended Data Fig. 1a and Supplementary Table 1). Of these, 21 samples and their matched normal counterparts underwent WES and constitute the discovery cohort. Another 39 TA-UC samples were subjected to ddPCR without matched normal counterparts and constitute the TAMARISK validation cohort. Formalin-fixed paraffin-embedded (FFPE) histopathology blocks were obtained, and H&E slides were reviewed by an expert pathologist to score tumor percentage and identify regions of high tumor content as well as regions of normal cells for isolation. Regions were macrodissected from five to ten 10-µm FFPE slides, and DNA was isolated from the excised tissue using the AllPrep DNA/RNA FFPE Isolation Kit (Qiagen, 80234) and the QIAcube according to the manufacturer’s protocols.

### Tamoxifen-associated uterine cancer from clinical databases

We identified a TA-UC clinical genomic data cohort by querying cancer registry data at the DFCI. We crossed the diagnosis of UC with the occurrence of breast cancer and tamoxifen treatment, searching for patients who had UC genotype data from the OncoPanel platform^70^. We identified an overall number of 120 patients, of whom 21 women had primary TA-UC (Extended Data Fig. 6c and Supplementary Tables 1 and 8), diagnosed between 2010 and 2022. A second TA-UC clinical genomic data cohort was obtained using the Caris Life Sciences internal cBioPortal, searching for patients treated with tamoxifen for breast cancer who were later diagnosed with UC. A total of 69 patients were identified, of whom 47 met the criteria for TA-UC, with diagnoses between 2015 and 2023 (Supplementary Table 1 and Extended Data Fig. 6g). Two de novo UC control sets were also identified using the Caris Life Sciences cBioPortal instance: (1) 8,258 patients with primary UC and no prior breast cancer diagnosis and (2) 569 patients with a history of breast cancer but no tamoxifen treatment and primary UC negative for homologous recombination deficiency, identified by the absence of *BRCA1* and *BRCA2* driver mutations and/or a low genomic scar score^71^. Genotype data were obtained as previously described^72,73^. We assessed potential overlap between the two TA-UC clinicogenomic datasets by comparing de-identified clinical variables, including date of UC diagnosis, age at UC diagnosis, histological UC type and prior breast cancer diagnosis. No overlap was found between patients in the two datasets.

### Whole-exome sequencing

Whole-exome capture was performed from tumor and normal DNA at the Broad Institute. DNA was quantified in triplicate using a standardized PicoGreen dsDNA Quantitation Reagent (Invitrogen) assay. The quality control identification check was performed using fingerprint genotyping of 95 common SNVs by Fluidigm Genotyping (Fluidigm). Samples were plated at a concentration of 2 ng µl^−1^ and a volume of 50 µl into matrix tubes, which allowed for positive barcode tracking throughout processing. Samples were sheared using a Broad-developed protocol optimized for a size distribution of ~180 bp. Library construction was performed using the KAPA Library Prep kit with palindromic forked adaptors from Integrated DNA Technologies. Libraries were pooled before hybridization. Hybridization and capture were performed using the relevant components of Illumina’s Rapid Capture Enrichment Kit, with a 37-Mb target. All library construction, hybridization and capture steps were automated on the Agilent Bravo liquid-handling system. After post-capture enrichment, library pools were quantified using qPCR, normalized to 2 nM and denatured using 0.1 M NaOH on the Hamilton STARlet. Flow cell cluster amplification and sequencing were performed according to the manufacturer’s protocols (Illumina) on either the HiSeq 2000 version 3 or HiSeq 2500 runs and used sequencing-by-synthesis kits to produce 76-bp paired reads. The target coverage was 150× mean target coverage for each tumor sample and 60× mean target coverage for each normal sample.

### Genomic data alignment and quality control

Data derived from WES were processed using established analytical tools within the Firehose platform (http://www.broadinstitute.org/cancer/cga/Firehose), which was later replaced with a cloud-based platform (FireCloud, Terra) operating on top of the Google Cloud Platform^74^. These platforms allow for coordinated and reproducible analysis of datasets using analytical pipelines. For each sample, the Picard data processing pipeline (version 2.9.2; http://broadinstitute.github.io/picard/) combines data from multiple libraries and flow cell runs into a single BAM file. Sequencing reads were aligned to the hg19 human genome build using BWA (http://bio-bwa.sourceforge.net). All sample pairs of tumor and normal genotypes were subjected to testing the level of cross-contamination using ContEst version 4 (ref. ^75^). We calculated the mean sequencing coverage for gene exonic regions using the DepthOfCoverage function from GATK version 4.1.6.0.

### Somatic mutation analysis

For each tumor–normal pair, somatic SNVs were called using MuTect (version 1)^76^ and small insertions and deletions (indels) with Strelka (version 2.9.0)^77^. These SNVs and indels were annotated using Oncotator (version 1.9.9.0)^78^. We excluded false-positive SNVs failing the following filters (version 25): (1) the OxoG filter^79^, which filters sequencing artifacts that are caused by oxidative damage to guanine during shearing in library preparation based on the read pair orientation bias, (2) the FFPE filter^80^, which filters sequencing artifacts caused by formaldehyde-induced deamination of cytosine based on the read pair orientation bias and (3) a mutational panel of normals^81^ built from FFPE samples sequenced using the same target regions, allowing us to filter the remaining potential sequencing artifacts as well as germline sites missed in the matched normal tissue. To recover SNVs lost to tumor-in-normal (TiN) contamination from adjacent tissue controls, we applied deTiN (version 3.0)^82^. In search for the presence of additional mutations (previously observed in TCGA de novo UCs) in the genes *ESR1*, *ESR2*, *PIK3CA*, *PIK3R1* and *PTEN*, we applied a ‘force-calling’ method (version 2)^83^, which calculates the number of reads supporting an alternate allele at predefined genomic coordinates. Manual review of mutations was performed using the Integrative Genomics Viewer^84^, and SNVs were filtered due to the following reasons: (1) low allelic fraction (AF) mutations, (2) mutations with orientation bias, (3) mutations called on reads that also contained indels and (4) mutations called in regions with poor mapping. Further downstream analysis was restricted to nonsynonymous mutations, ignoring mutations classified as 3′ UTR, 5′ UTR, IGR, intron, lincRNA, RNA or silent.

### Mutational significance analysis

Significance analysis of recurrently mutated genes was performed using MutSig2CV (version 3.11 with ‘gene_min_frac_coverage_required’ set to 0.02), which detects genes with a higher-than-expected SNV frequency or an unexpected pattern of SNVs^85^. Significantly mutated genes were defined as genes with *Q* < 0.1 using the method of Benjamini and Hochberg^86^ to convert final *P* values to false discovery rate *Q* values. In addition, we used restricted hypothesis testing (as we have done previously^87^) using a panel of 113 previously published UC genes (Supplementary Table 4)^29–31,34^ to identify additional recurrently mutated genes. Because our aim was not to perform a de novo discovery of driver genes in the control cohort, we restricted the MutSig2CV analysis in the TCGA sample set of de novo UCs to the above panel of known UC drivers. We tested for mutual exclusivity and co-occurrence on a patient mutational level by applying Fisher’s exact test.

### Somatic copy number analysis

GATK4’s copy number variant discovery pipeline was used to analyze read coverage and detect copy number and allelic copy number alterations (release 4.1.6.0; variances of Gaussian kernel for copy ratio segmentation and allele fraction segmentation were set to 0.175 and 0.2, respectively). A copy number panel of normals used normal samples with low TiN to normalize the read depth at each capture probe. In addition, we tagged and removed copy number segments caused by potential germline events by comparing break points and reciprocal overlaps. Manual review of SCNAs was performed using the Integrative Genomics Viewer (version 2.16.2)^84^.

### Copy number significance analysis

GISTIC2.0 (version 2.03.23)^36^ was applied to detect significantly amplified or deleted SCNAs across a cohort using a threshold of *Q* < 0.25. Peaks were annotated with genes from the Cancer Gene Census^88^. *G* scores were assigned to each peak considering the amplitude of the alteration and the frequency of its occurrence across specimens.

### ABSOLUTE, phylogeny and timing analyses

ABSOLUTE version 1.5 (ref. ^89^) was used to estimate purity (that is, the percentage of tumor cells in the cancer sample), ploidy (that is, the average copy number across the cancer genome), absolute copy numbers and WGD status for each tumor sample. ABSOLUTE solutions were manually curated. To determine whether mutations are clonal (that is, present in all tumor cells), we used the CCF of each mutation provided by ABSOLUTE (mutations with an estimated CCF ≥ 0.95 are considered clonal; mutations with lower CCFs are considered subclonal).

To analyze the phylogenetic relationship between tumor cell populations within a tumor, we used PhylogicNDT (version 35)^57,58^, an N-dimensional Bayesian clustering framework based on mixtures of Dirichlet processes, in which the number of clusters is inferred over many Markov chain Monte Carlo iterations. Clusters of mutations with consistent CCF were used to determine the phylogenetic tree that best represents the clonal evolution. The tumor developmental trajectory was probabilistically determined, allowing us to order and estimate relative timing of clonal events and WGD (SinglePatientTiming and PhylogicNDT LeagueModel for ordering of events across a sample set).

### Prediction of microsatellite instability

MSI was predicted using MSIdetect (version 2) as described before^90^. In short, MSIdetect assigns a probability for every read from a sequenced sample as coming from a tumor with MSI or an MSS tumor and aggregates it over all reads to generate an MSI score. Because the MSI score varies between sequencing platforms, we used normal samples to set the threshold between MSI and MSS patients.

### Mutational signature analysis

SignatureAnalyzer (version 0.0.8)^91^, a Bayesian nonnegative matrix factorization method, was used to extract mutational signatures from SNVs by considering the 96 single-base substitutions within the trinucleotide sequence context. Signatures were then compared with previously described signatures in COSMIC version 3 (https://cancer.sanger.ac.uk/cosmic/signatures). We also applied supervised Bayesian nonnegative matrix factorization implemented for GPUs^92^ specifying a set of 13 expected COSMIC version 3 signatures (aging: SBS1, SBS5; MSI: SBS6, SBS14, SBS15, SBS20, SBS21, SBS26, SBS44; POLE: SBS10a, SBS10b, SBS14) to infer their contributions.

### Analysis of molecular subtypes

To replicate the molecular subtype analysis from TCGA^29^, we used the following approach. First, samples were assigned to the POLE subtype if they had POLE exonuclease domain mutations and associated mutational signatures (COSMIC signatures SBS10a, SBS10b and SBS14). Next, samples with MSI (MSI subtype) were classified using MSIdetect and then validated by the presence of mutational signatures associated with it^93^ (COSMIC signatures SBS6, SBS14, SBS15, SBS20, SBS21, SBS26 and SBS44). The remaining samples were categorized into two groups (CIN and genomically stable) based on their copy number pattern. As described previously^94^, the CIN subtype is characterized by a high rate of deletions. We calculated the fraction of the genome that was deleted by including copy number events of all lengths with a copy number change larger than a given threshold (*R*_1_ = 0.36). Because impure samples have a smaller change in copy number than samples with high purity, the threshold was normalized by the inferred purity. Samples were categorized as CIN when the fraction of the deleted genome was larger than a given threshold (*R*_2_ = 0.034). Molecular subtyping was applied to TA-UC and de novo TCGA UC where we did not have previous annotations for molecular subtypes; published molecular subtypes were used for endometrial carcinomas^29^. Above thresholds were determined by analyzing TCGA Uterine Corpus Endometrial Carcinoma data. ABSOLUTE purity data for TCGA samples were used from Taylor et al.^95^.

### Droplet digital PCR

ddPCR was used to detect hotspot mutations in the *PIK3CA* and *ESR1* genes using FFPE-derived DNA from (1) 19 TA-UCs that had undergone WES and had residual DNA and (2) an independent cohort of 39 TA-UC tumors. TaqMan PCR reaction mixtures were assembled from a 2× ddPCR master mix (Bio-Rad) and custom 40× TaqMan probes or primers made specific for each assay (Thermo Fisher Scientific). Assembled ddPCR reaction mixture (25 μl), which included either 5 μl DNA sample or water as a no-template control, was loaded into wells of a 96-well PCR plate. The heat-sealed PCR plate was subsequently loaded onto the Automated Droplet Generator (Bio-Rad). After droplet generation, the new 96-well PCR plate was heat sealed, placed on a conventional thermal cycler and amplified to the end point. After PCR, the 96-well PCR plate was read on the QX100 Droplet Reader (Bio-Rad). The primers applied in this analysis have been validated and described previously^96,97^. Analysis of the ddPCR data was performed with QuantaSoft analysis software (Bio-Rad) that accompanied the droplet reader. We calculated the AF (in percent) as AF = (count mutant droplets)(count wild-type droplets + count mutant droplets)^−1^ × 100 and applied a cutoff of >2% AF to reduce FFPE-associated false positives.

### Published human datasets

For comparison of histologic subtypes, research data from 40,587 unique UC tumors diagnosed between 1973 and 2015 were obtained from the SEER9 registries (data released April 2018, based on the November 2017 submission). Tumors were distributed among the nine SEER registries as follows: 17% from San Francisco–Oakland, 13% from Connecticut, 16% from Metropolitan Detroit, 4% from Hawaii, 16% from Iowa, 5% from New Mexico, 16% from Seattle, 6% from Utah and 7% from Metropolitan Atlanta. To match the time frame of our cohorts, only tumors diagnosed between 1983 and 2002 were included. Primary site UCs (ICD-0-2 codes C54.0–C54.3, C54.8–C54.9, C55.9) classified as malignant (ICD-0-3 code 3) were used. To conservatively restrict the dataset to de novo UCs, women with breast cancer history (ICD-0-2 codes C50.0–C50.6, C50.8–C50.9) were excluded, as some may have developed TA-UC following prior tamoxifen treatment. Histologic subtypes were categorized as follows: endometrioid endometrial adenocarcinoma (8050, 8140, 8143, 8210, 8211, 8260, 8261, 8262, 8263, 8380, 8381, 8382, 8383, 8384, 8560, 8570); clear cell (8310) and serous adenocarcinoma (8441, 8460, 8461); mixed (8255, 8323); malignant Mullerian mixed tumors or carcinosarcoma (8950, 8951, 8980, 8981); and sarcoma (8890, 8891, 8896, 8930, 8931, 8935, 8933, 8800, 8801, 8802, 8803, 8804, 8805).

Additionally, we used 554 whole-exome sequenced primary de novo UC samples from TCGA for which data on absolute copy number, SNVs, survival, histological subtype and other clinical variables were available from the MC3 TCGA project^81^ (Extended Data Fig. 3a). CCFs were identified from the ABSOLUTE-annotated MAF file of the Pan-Cancer TCGA project and Haradhvala et al.^93^ for 536 of 554 TCGA UC samples. Copy number data were retrieved for a whitelisted set of 544 of 554 tumors. We applied the following criteria to identify de novo TCGA UC samples and exclude prior tamoxifen use: (1) 54 patients were annotated as having no prior tamoxifen use, (2) 482 patients had no prior diagnosis of a malignancy, (3) 16 patients had a prior diagnosis of cancer other than a breast malignancy and (4) two patients were diagnosed with breast cancer, but detailed treatment information excluded prior tamoxifen use. This set of 554 TCGA samples was composed of the following histological types: (1) a sample set containing 371 endometrioid endometrial adenocarcinomas, 96 serous endometrial adenocarcinomas and 19 mixed serous and endometrioid tumors from TCGA Uterine Corpus Endometrial Carcinoma^29^, (2) 52 uterine carcinosarcomas from TCGA-UCS^30^ and (3) 16 uterine sarcomas from TCGA-SARC^31^. For 508 of these patients, height and weight data were available, and BMI was calculated by dividing body weight in kilograms by height in meters squared (kg m^−2^).

In addition, we searched TCGA annotation files and pathology reports to identify patients with UC and a previous history of tamoxifen use and identified two such patients with TA-UC in the TCGA cohort (TCGA TA-UCs TCGA-BG-A0MS and TCGA-IW-A3M6), who were analyzed separately.

Another set of 130 de novo UC specimens (111 endometrioid endometrial adenocarcinomas, 13 serous endometrial adenocarcinomas, three clear cell carcinomas, three not further defined) with available data on BMI as determined above were used from the Clinical Proteomic Tumor Analysis Consortium^94^.

We also included 834 primary de novo UC specimens with consistent histology and available mutation data from unique patients from the AACR GENIE Project (version 13.0)^32^ that originated from the DFCI. Patients with TA-UC (as identified at the DFCI and described above) were excluded. The final set included 527 endometrioid and mixed endometrial adenocarcinomas; 165 serous and clear cell tumors; 93 carcinosarcomas; and 49 leiomyosarcomas.

Although overlap between the US de novo UC cohorts (TCGA, GENIE, CARIS) is highly unlikely due to differences in sample origin, diagnosis data, histology and age at diagnosis, the use of de-identified data means that we cannot completely exclude this possibility, which is a limitation of the study.

In addition, somatic mutation sets from the following noncancerous FFPE tissue types were used: (1) normal endometrial tissue^62^, (2) endometriosis^63,64^ and (3) atypical hyperplasia^65,66^.

Finally, we also included histological subtype data from a set of 161 TAMARISK patients with de novo UC^28^ diagnosed after breast cancer but without prior use of tamoxifen.

### Statistics and reproducibility

Statistical analysis and visualization were performed using R (version 4.1.1) in an RStudio environment and Julia (version 1.7.3) in a Jupyter environment. To determine significance, we used Fisher’s exact test (with Monte Carlo simulation for tables larger than 2 × 2, using 10^6^ iterations), the *t*-test and the Wilcoxon rank-sum test, all two sided unless otherwise indicated. Multiple-hypothesis testing was performed using the method of Benjamini and Hochberg^86^, which converted the final *P* values to false discovery rate *Q* values; *Q* < 0.1 was considered significant. The strength of associations between variables was analyzed using Pearson’s correlation. Two-sided stratified Fisher’s exact test was used to control for potential confounding variables when analyzing mutation frequency data across multiple subgroups (or strata), providing a combined *P* value calculated across the strata, with zero-marginal tables excluded from the calculation^98,99^. No statistical method was used to predetermine sample size. No data were excluded from the analyses. Randomization and blinding were not applicable, as this study involved retrospective analysis of genomic and clinical data.

### Power calculations

We assessed the statistical power to detect differences in driver gene mutation frequencies (either higher or lower) between the TA-UC and de novo UC sample sets given the observed sample sizes in both the WES discovery cohort and the WES validation subtypes. We identified powered genes by computing Bonferroni-corrected two-sided optimal Fisher’s exact test *P* values across all possible 2 × 2 contingency tables, maintaining the same marginal totals but allowing zero counts. For each configuration, we calculated *P* values, focusing on the smallest *P* value as an indication of the extreme case in which the effect size is close to or equal to zero. A Bonferroni-corrected optimal *P* value of <0.05 was considered a powered test. We also calculated the power to identify driver genes that are significantly less mutated in the TA-UC discovery cohort by computing *P* values from one-sided Fisher’s exact tests for the different frequencies. Genes at a threshold of *P* < 0.05 can potentially be considered significantly less mutated in the TA-UC discovery cohort, as they are mutated in at least 76 de novo TCGA UC samples.

### Analysis of human expression data

We used previously published^100^ gene expression levels from Affymetrix U95A Human Genome arrays of enriched human-derived endometrial cells that were short-term cultured with either E2 (100 nM) or tamoxifen (5 µM) for 3 h. After removal of one outlier sample (GSM65291), we performed quantile normalization followed by differential gene expression using limmaVoom^101^ (version 3.50.0), focusing on genes in the KEGG PATHWAY Database, estrogen response genes from the hallmark gene sets and genes in the AKT–mTOR oncogenic signature gene sets (all from GSEA). Pathway analysis was carried out using Enrichr (https://maayanlab.cloud/Enrichr/)^102^, the NCI–Nature Pathway Interaction Database^103^ and differentially expressed genes with a cutoff of |log_2_ (FC)| > log_2_ (1.5) and *Q* value < 0.01.

### In vivo mouse study

All mice were maintained in accordance with local guidelines, and therapeutic interventions were approved by the Animal Care and Use Committee of the DFCI (protocol 08-023). To mimic the postmenopausal condition that is typically observed in patients with TA-UC, 20 C57BL/6 female mice (Jackson Laboratory) were oophorectomized after sexual maturity (6–7 weeks) to allow for proper uterine development. Oophorectomy also circumvented the ER-dependent endometrial changes that occur during the estrous cycle, which could confound the interpretation of results. As the hormone E2, a major female sex hormone produced during the estrous cycle, binds to ER and increases cell proliferation, we used exogenous E2 as a positive control. Mice were randomized (*n* = 5 per arm) to E2 (0.01 mg per pellet, 60-d release), vehicle control (E2 deprived), tamoxifen (Sigma, in 20% ethanol in corn oil, 0.5 mg per mouse per day, subcutaneous injection, comparable to the concentration seen in humans^104^) or tamoxifen plus alpelisib (Selleckchem, in 30% PEG 400 + 0.5% Tween-80 + 5% propylene glycol, 30 mg per kg per day, oral gavage) for 30 d. At the end of the study, mice were euthanized, and uterine horns were collected.

### Mouse tissue collection and processing

Mouse uterine horns were collected from five mice per cohort, as reported by De Clercq et al.^105^. Samples were allocated for downstream applications as follows: (1) single-cell suspensions were prepared and used to isolate epithelial and stromal cell populations. For the E2, tamoxifen and tamoxifen-plus-alpelisib groups, three mice per condition were used; in the vehicle control group, five mice were processed to obtain sufficient material despite the minuscule size of the uteri in this condition. (2) FFPE samples for IHC were prepared from three mice (E2), five mice (tamoxifen, tamoxifen plus alpelisib) and two mice (vehicle control, in which sample collection was limited by the miniscule size of the uterine horns, a consequence of oophorectomy without hormonal supplementation, and by fibrosis secondary to the surgical procedure).

### Immunohistochemistry

For immunohistochemical detection, samples were stained with primary antibodies and incubated with anti-mouse (G21040, Invitrogen) or anti-rabbit (G21234, Invitrogen) antibodies (both at a 1:2,000 dilution) for 50 min at room temperature. Samples were stained with the DAB (3,3′-diaminobenzidine) colorimetric substrate and counterstained with hematoxylin. The following primary antibodies were used: anti-ER-α (06-938, 1:1,000, Millipore), anti-phospho-IR/IGF1R Tyr1162/Tyr1163 (44-804, 1:500, Invitrogen), anti-Ki-67 (ab15580, 1:1,000, Abcam), anti-phospho-AKT Thr308 (ab81283, 1:50, Abcam) and anti-phospho-S6 Ser240/Ser244 (2215, 1:500, Cell Signaling).

Numbers of ducts per mouse were counted in six distinct sections using a 20× high-power field. The length (in µm) of endometrial epithelial cells per mouse was measured in six sections using five distinct regions of the internal lumen. IHC images were analyzed with QuPath version 0.2.0 software (https://qupath.github.io/). IHC staining was quantified as the product of percent positive cells per section × staining intensity in optical density (*H* score). Statistical analyses for immunohistochemical studies were performed in GraphPad Prism version 9.0 (GraphPad Software) using one-way ANOVA.

### Messenger RNA in situ hybridization

In situ hybridization was performed with the RNAscope Intro Pack for Multiplex Fluorescent Reagent Kit v2-Mm from Advanced Cell Diagnostics according to the manufacturer’s protocol. Briefly, FFPE sections were deparaffinized with xylene and rehydrated with alcohol. The sections were hybridized at 40 °C for 2 h with the RNAscope Probe-Mm-Igf1 that is specific for mouse *Igf1* mRNA (Advanced Cell Diagnostics), and the signal was visualized with RNAscope fluorescent reagents. Sections were counterstained with ProLong Gold Antifade Reagent (Life Technologies) before dehydrating, and coverslips were affixed with Permount (Thermo Fisher Scientific). Images were acquired with a Leica SP8X STED/confocal microscope using Leica Application Suite X (version 3.7) acquisition software. Images were acquired as *Z* stacks (1 µm) using the Piezo Z stage.

### RNA extraction and quantitative PCR with reverse transcription

Total RNA was isolated using TRIzol (Life Technologies) and the RNeasy Mini Kit (Qiagen) according to the manufacturer’s instructions. To test the purity of epithelial cells, we used quantitative PCR with reverse transcription and primers summarized in Supplementary Table 15. mRNA was retrotranscribed using the High-Capacity cDNA Reverse Transcription Kit (Applied Biosystem), and detection was accomplished using the Roche LightCycler 480 Real-time PCR system in combination with the Power SYBR Green PCR Master Mix (Life Technologies).

### RNA sequencing

RNA-seq libraries were made after enrichment with oligo(dT) beads. First, mRNA was randomly fragmented by adding fragmentation buffer. Next, cDNA was synthesized using mRNA template and random hexamer primers, after which a custom second-strand synthesis buffer (Illumina), dNTPs, RNase H and DNA polymerase I were added to initiate second-strand synthesis. After a series of terminal repair, A ligation and sequencing adaptor ligation, the double-stranded cDNA library was completed through size selection and PCR enrichment. Samples were sequenced on an Illumina NextSeq 500 instrument (libraries generated and sequencing performed at Novogene).

### RNA sequencing analysis

RNA-seq analysis was performed using the VIPER analysis pipeline (version 1.41.0)^106^. Alignment to the hg19 human genome was accomplished using STAR version 2.7.0f followed by transcript assembly using cufflinks version 2.2.1 (ref. ^107^) and RSeQC version 2.6.2 (ref. ^108^). Differential expression analysis was carried out using DESeq2 version 1.18.1 (ref. ^109^). Pathway analysis was carried out using Enrichr (https://maayanlab.cloud/Enrichr/) and applying MsigDB oncogenic signatures^102^.

### Reporting summary

Further information on research design is available in the Nature Portfolio Reporting Summary linked to this article.

## Online content

Any methods, additional references, Nature Portfolio reporting summaries, source data, extended data, supplementary information, acknowledgements, peer review information; details of author contributions and competing interests; and statements of data and code availability are available at 10.1038/s41588-025-02308-w.

## Supplementary information

Supplementary Information Supplementary Figs. 1 and 2 and Notes 1–7.

Reporting Summary

Supplementary Tables 1–15 See specific legends in the first tab ‘Table Explanations’.

## Data Availability

TCGA pan-cancer data are available through a data portal: https://gdc.cancer.gov/node/905/; https://gdc.cancer.gov/about-data/publications/pancanatlas. In compliance with the data access policy, most data are in an open tier that does not require access approval. Some data files with potentially identifying information and underlying sequencing data are controlled-access data and may be hosted at dbGaP. Researchers will need to apply to the TCGA Data Access Committee via dbGaP (https://dbgap.ncbi.nlm.nih.gov/aa/wga.cgi?page=login) to request access. Clinical Proteomic Tumor Analysis Consortium endometrial cancer mutation data are available from the Genomic Data Commons (https://gdc.cancer.gov/) or upon request from dbGaP (https://www.ncbi.nlm.nih.gov/gap/, phs001287). SEER data are available through a data portal (https://seer.cancer.gov/data/) after data use agreement forms have been signed. The Affymetrix U95A Human Genome arrays of enriched human-derived endometrial cells can be accessed at the Gene Expression Omnibus via GSE3013. Data from the GENIE database can be found on the Sage Bionetworks portal (https://www.synapse.org/#!Synapse:syn7222066/wiki/405659). To request access to protected GENIE data, researchers need to apply to dbGaP for access (study accession phs001337). Analyses in this paper also used published datasets that are available from the corresponding studies, which are referenced where relevant. WES data of TA-UCs are available through the EGA; the accession number is EGAS00001006453 (https://www.ega-archive.org/studies/EGAS00001006453). Mouse endometrial epithelial RNA-seq data are available at the Gene Expression Omnibus through GSE179647 (https://www.ncbi.nlm.nih.gov/geo/query/acc.cgi?acc=GSE179647). The Caris datasets generated and/or analyzed during the current study are available upon reasonable request. De-identified sequencing data are owned by Caris Life Sciences and cannot be publicly shared without a data usage agreement. Qualified researchers can apply for access to these summarized data by contacting J. Xiu (jxiu@carisls.com) and signing a data usage agreement. MsigDB oncogenic signatures, KEGG PATHWAY database, estrogen response genes from the hallmark gene sets and genes in the AKT–mTOR oncogenic signature gene sets are from https://www.gsea-msigdb.org/gsea; the NCI–Nature Pathway Interaction Database can be found at https://www.ndexbio.org/.

## Code availability

No custom code was used. All software packages and analysis code are publicly available, and the relevant sources are cited in Methods.

## References

[1] Kuijk, E., Kranenburg, O., Cuppen, E. & Van Hoeck, A. Common anti-cancer therapies induce somatic mutations in stem cells of healthy tissue. *Nat. Commun.* **13**, 5915 (2022). 36207433 10.1038/s41467-022-33663-5 PMC9546852

[2] Carthew, P. et al. DNA damage as assessed by ^32^P-postlabelling in three rat strains exposed to dietary tamoxifen: the relationship between cell proliferation and liver tumour formation. *Carcinogenesis* **16**, 1299–1304 (1995). 7788846 10.1093/carcin/16.6.1299

[3] Carthew, P. et al. Cumulative exposure to tamoxifen: DNA adducts and liver cancer in the rat. *Arch. Toxicol.* **75**, 375–380 (2001). 11570696 10.1007/s002040100244

[4] Busch, H. Adducts and tamoxifen. *Semin. Oncol.* **24**, S1-98–S1-104 (1997). 9045322

[5] Hernandez-Ramon, E. E. et al. Tamoxifen–DNA adduct formation in monkey and human reproductive organs. *Carcinogenesis* **35**, 1172–1176 (2014). 24501327 10.1093/carcin/bgu029 PMC4004208

[6] Andersson, H., Helmestam, M., Zebrowska, A., Olovsson, M. & Brittebo, E. Tamoxifen-induced adduct formation and cell stress in human endometrial glands. *Drug Metab. Dispos.* **38**, 200–207 (2010). 19812351 10.1124/dmd.109.029488

[7] Kim, S. Y. et al. Formation of tamoxifen–DNA adducts in human endometrial explants exposed to α-hydroxytamoxifen. *Chem. Res. Toxicol.* **18**, 889–895 (2005). 15892583 10.1021/tx050019l

[8] Cole, M. P., Jones, C. T. & Todd, I. D. A new anti-oestrogenic agent in late breast cancer. An early clinical appraisal of ICI46474. *Br. J. Cancer* **25**, 270–275 (1971). 5115829 10.1038/bjc.1971.33 PMC2008453

[9] Fisher, B. et al. Adjuvant chemotherapy with and without tamoxifen in the treatment of primary breast cancer: 5-year results from the National Surgical Adjuvant Breast and Bowel Project Trial. *J. Clin. Oncol.* **4**, 459–471 (1986). 2856857 10.1200/JCO.1986.4.4.459

[10] Fisher, B. et al. Tamoxifen for prevention of breast cancer: report of the National Surgical Adjuvant Breast and Bowel Project P-1 Study. *J. Natl Cancer Inst.* **90**, 1371–1388 (1998). 9747868 10.1093/jnci/90.18.1371

[11] Early Breast Cancer Trialists’ Collaborative Group. Aromatase inhibitors versus tamoxifen in early breast cancer: patient-level meta-analysis of the randomised trials. *Lancet* **386**, 1341–1352 (2015). 26211827 10.1016/S0140-6736(15)61074-1

[12] Sparano, J. A. et al. Adjuvant chemotherapy guided by a 21-gene expression assay in breast cancer. *N. Engl. J. Med.* **379**, 111–121 (2018). 29860917 10.1056/NEJMoa1804710 PMC6172658

[13] Johnston, S. R. D. et al. Abemaciclib combined with endocrine therapy for the adjuvant treatment of HR^+^, HER2^−^, node-positive, high-risk, early breast cancer (monarchE). *J. Clin. Oncol.* **38**, 3987–3998 (2020). 32954927 10.1200/JCO.20.02514 PMC7768339

[14] Fornander, T. et al. Adjuvant tamoxifen in early breast cancer: occurrence of new primary cancers. *Lancet* **1**, 117–120 (1989). 2563046 10.1016/s0140-6736(89)91141-0

[15] Bernstein, L. et al. Tamoxifen therapy for breast cancer and endometrial cancer risk. *J. Natl Cancer Inst.* **91**, 1654–1662 (1999). 10511593 10.1093/jnci/91.19.1654

[16] Bergman, L. et al. Risk and prognosis of endometrial cancer after tamoxifen for breast cancer. Comprehensive Cancer Centres’ ALERT Group. Assessment of liver and endometrial cancer risk following tamoxifen. *Lancet* **356**, 881–887 (2000). 11036892 10.1016/s0140-6736(00)02677-5

[17] Fisher, B. et al. Endometrial cancer in tamoxifen-treated breast cancer patients: findings from the National Surgical Adjuvant Breast and Bowel Project (NSABP) B-14. *J. Natl Cancer Inst.* **86**, 527–537 (1994). 8133536 10.1093/jnci/86.7.527

[18] Swerdlow, A. J., Jones, M. E. & British Tamoxifen Second Cancer Study Group. Tamoxifen treatment for breast cancer and risk of endometrial cancer: a case–control study. *J. Natl Cancer Inst.* **97**, 375–384 (2005). 15741574 10.1093/jnci/dji057

[19] Cuzick, J. et al. Tamoxifen for prevention of breast cancer: extended long-term follow-up of the IBIS-I breast cancer prevention trial. *Lancet Oncol.* **16**, 67–75 (2015). 25497694 10.1016/S1470-2045(14)71171-4 PMC4772450

[20] Fisher, B. et al. Tamoxifen for the prevention of breast cancer: current status of the National Surgical Adjuvant Breast and Bowel Project P-1 study. *J. Natl Cancer Inst.* **97**, 1652–1662 (2005). 16288118 10.1093/jnci/dji372

[21] Davies, C. et al. Long-term effects of continuing adjuvant tamoxifen to 10 years versus stopping at 5 years after diagnosis of oestrogen receptor-positive breast cancer: ATLAS, a randomised trial. *Lancet* **381**, 805–816 (2013). 23219286 10.1016/S0140-6736(12)61963-1 PMC3596060

[22] Shang, Y. & Brown, M. Molecular determinants for the tissue specificity of SERMs. *Science* **295**, 2465–2468 (2002). 11923541 10.1126/science.1068537

[23] Korach, K. S. Insights from the study of animals lacking functional estrogen receptor. *Science* **266**, 1524–1527 (1994). 7985022 10.1126/science.7985022

[24] Couse, J. F. & Korach, K. S. Estrogen receptor null mice: what have we learned and where will they lead us? *Endocr. Rev.* **20**, 358–417 (1999). 10368776 10.1210/edrv.20.3.0370

[25] Davies, R. et al. Tamoxifen causes gene mutations in the livers of lambda/*lacI* transgenic rats. *Cancer Res.* **57**, 1288–1293 (1997). 9102215

[26] Brown, K. Is tamoxifen a genotoxic carcinogen in women? *Mutagenesis* **24**, 391–404 (2009). 19505894 10.1093/mutage/gep022

[27] Fles, R. et al. Genomic profile of endometrial tumors depends on morphological subtype, not on tamoxifen exposure. *Genes Chromosomes Cancer* **49**, 699–710 (2010). 20544844 10.1002/gcc.20781

[28] Hoogendoorn, W. E. et al. Prognosis of uterine corpus cancer after tamoxifen treatment for breast cancer. *Breast Cancer Res. Treat.* **112**, 99–108 (2008). 18064567 10.1007/s10549-007-9823-1

[29] Levine, D. A. et al. Integrated genomic characterization of endometrial carcinoma. *Nature* **497**, 67–73 (2013). 23636398 10.1038/nature12113 PMC3704730

[30] Cherniack, A. D. et al. Integrated molecular characterization of uterine carcinosarcoma. *Cancer Cell* **31**, 411–423 (2017). 28292439 10.1016/j.ccell.2017.02.010 PMC5599133

[31] Cancer Genome Atlas Research Network. Comprehensive and integrated genomic characterization of adult soft tissue sarcomas. *Cell* **171**, 950–965 (2017). 29100075 10.1016/j.cell.2017.10.014 PMC5693358

[32] AACR Project GENIE Consortium. AACR Project GENIE: powering precision medicine through an international consortium. *Cancer Discov.* **7**, 818–831 (2017). 28572459 10.1158/2159-8290.CD-17-0151 PMC5611790

[33] Alexandrov, L. B. et al. The repertoire of mutational signatures in human cancer. *Nature* **578**, 94–101 (2020). 32025018 10.1038/s41586-020-1943-3 PMC7054213

[34] Gibson, W. J. et al. The genomic landscape and evolution of endometrial carcinoma progression and abdominopelvic metastasis. *Nat. Genet.* **48**, 848–855 (2016). 27348297 10.1038/ng.3602 PMC4963271

[35] Chang, M. T. et al. Identifying recurrent mutations in cancer reveals widespread lineage diversity and mutational specificity. *Nat. Biotechnol.* **34**, 155–163 (2016). 26619011 10.1038/nbt.3391 PMC4744099

[36] Mermel, C. H. et al. GISTIC2.0 facilitates sensitive and confident localization of the targets of focal somatic copy-number alteration in human cancers. *Genome Biol.* **12**, R41 (2011). 21527027 10.1186/gb-2011-12-4-r41 PMC3218867

[37] Zhang, Y. et al. A pan-cancer proteogenomic atlas of PI3K/AKT/mTOR pathway alterations. *Cancer Cell* **31**, 820–832 (2017). 28528867 10.1016/j.ccell.2017.04.013 PMC5502825

[38] Dashti, S. G. et al. Adiposity and breast, endometrial, and colorectal cancer risk in postmenopausal women: quantification of the mediating effects of leptin, C-reactive protein, fasting insulin, and estradiol. *Cancer Med.* **11**, 1145–1159 (2022). 35048536 10.1002/cam4.4434 PMC8855919

[39] Kaaks, R., Lukanova, A. & Kurzer, M. S. Obesity, endogenous hormones, and endometrial cancer risk: a synthetic review. *Cancer Epidemiol. Biomarkers Prev.* **11**, 1531–1543 (2002). 12496040

[40] Schmandt, R. E., Iglesias, D. A., Co, N. N. & Lu, K. H. Understanding obesity and endometrial cancer risk: opportunities for prevention. *Am. J. Obstet. Gynecol.* **205**, 518–525 (2011). 21802066 10.1016/j.ajog.2011.05.042 PMC4264838

[41] Freeman, E. W., Sammel, M. D., Lin, H. & Gracia, C. R. Obesity and reproductive hormone levels in the transition to menopause. *Menopause* **17**, 718–726 (2010). 20216473 10.1097/gme.0b013e3181cec85d PMC2888623

[42] Onstad, M. A., Schmandt, R. E. & Lu, K. H. Addressing the role of obesity in endometrial cancer risk, prevention, and treatment. *J. Clin. Oncol.* **34**, 4225–4230 (2016). 27903150 10.1200/JCO.2016.69.4638 PMC5455320

[43] Smith, D. C., Prentice, R., Thompson, D. J. & Herrmann, W. L. Association of exogenous estrogen and endometrial carcinoma. *N. Engl. J. Med.* **293**, 1164–1167 (1975). 1186789 10.1056/NEJM197512042932302

[44] Ziel, H. K. & Finkle, W. D. Increased risk of endometrial carcinoma among users of conjugated estrogens. *N. Engl. J. Med.* **293**, 1167–1170 (1975). 171569 10.1056/NEJM197512042932303

[45] Yuan, T. L. & Cantley, L. C. PI3K pathway alterations in cancer: variations on a theme. *Oncogene* **27**, 5497–5510 (2008). 18794884 10.1038/onc.2008.245 PMC3398461

[46] Cheung, L. W. et al. High frequency of *PIK3R1* and *PIK3R2* mutations in endometrial cancer elucidates a novel mechanism for regulation of PTEN protein stability. *Cancer Discov.* **1**, 170–185 (2011). 21984976 10.1158/2159-8290.CD-11-0039 PMC3187555

[47] Fleming, C. A. et al. Meta-analysis of the cumulative risk of endometrial malignancy and systematic review of endometrial surveillance in extended tamoxifen therapy. *Br. J. Surg.* **105**, 1098–1106 (2018). 29974455 10.1002/bjs.10899

[48] André, F. et al. Alpelisib for *PIK3CA*-mutated, hormone receptor-positive advanced breast cancer. *N. Engl. J. Med.* **380**, 1929–1940 (2019). 31091374 10.1056/NEJMoa1813904

[49] Rodriguez, A. C., Blanchard, Z., Maurer, K. A. & Gertz, J. Estrogen signaling in endometrial cancer: a key oncogenic pathway with several open questions. *Horm. Cancer* **10**, 51–63 (2019). 30712080 10.1007/s12672-019-0358-9 PMC6542701

[50] Adesanya, O. O., Zhou, J., Samathanam, C., Powell-Braxton, L. & Bondy, C. A. Insulin-like growth factor 1 is required for G_2_ progression in the estradiol-induced mitotic cycle. *Proc. Natl Acad. Sci. USA* **96**, 3287–3291 (1999). 10077676 10.1073/pnas.96.6.3287 PMC15934

[51] Klotz, D. M., Hewitt, S. C., Korach, K. S. & Diaugustine, R. P. Activation of a uterine insulin-like growth factor I signaling pathway by clinical and environmental estrogens: requirement of estrogen receptor-α. *Endocrinology* **141**, 3430–3439 (2000). 10965916 10.1210/endo.141.9.7649

[52] Aronica, S. M. & Katzenellenbogen, B. S. Stimulation of estrogen receptor-mediated transcription and alteration in the phosphorylation state of the rat uterine estrogen receptor by estrogen, cyclic adenosine monophosphate, and insulin-like growth factor-I. *Mol. Endocrinol.* **7**, 743–752 (1993). 7689695 10.1210/mend.7.6.7689695

[53] Martin, M. B. et al. A role for Akt in mediating the estrogenic functions of epidermal growth factor and insulin-like growth factor I. *Endocrinology* **141**, 4503–4511 (2000). 11108261 10.1210/endo.141.12.7836

[54] Kashima, H. et al. Autocrine stimulation of IGF1 in estrogen-induced growth of endometrial carcinoma cells: involvement of the mitogen-activated protein kinase pathway followed by up-regulation of cyclin D1 and cyclin E. *Endocr. Relat. Cancer* **16**, 113–122 (2009). 18852162 10.1677/ERC-08-0117

[55] Cooke, P. S. et al. Stromal estrogen receptors mediate mitogenic effects of estradiol on uterine epithelium. *Proc. Natl Acad. Sci. USA* **94**, 6535–6540 (1997). 9177253 10.1073/pnas.94.12.6535 PMC21085

[56] Baxter, R. C. Signalling pathways involved in antiproliferative effects of IGFBP-3: a review. *Mol. Pathol.* **54**, 145–148 (2001). 11376125 10.1136/mp.54.3.145 PMC1187052

[57] Dentro, S. C. et al. Characterizing genetic intra-tumor heterogeneity across 2,658 human cancer genomes. *Cell* **184**, 2239–2254 (2021). 33831375 10.1016/j.cell.2021.03.009 PMC8054914

[58] Leshchiner, I. et al. Inferring early genetic progression in cancers with unobtainable premalignant disease. *Nat. Cancer* **4**, 550–563 (2023). 37081260 10.1038/s43018-023-00533-y PMC10132986

[59] van Leeuwen, F. E. et al. Risk of endometrial cancer after tamoxifen treatment of breast cancer. *Lancet* **343**, 448–452 (1994). 7905955 10.1016/s0140-6736(94)92692-1

[60] Berg, A. et al. Molecular profiling of endometrial carcinoma precursor, primary and metastatic lesions suggests different targets for treatment in obese compared to non-obese patients. *Oncotarget* **6**, 1327–1339 (2015). 25415225 10.18632/oncotarget.2675 PMC4359236

[61] Moore, L. et al. The mutational landscape of normal human endometrial epithelium. *Nature* **580**, 640–646 (2020). 32350471 10.1038/s41586-020-2214-z

[62] Lac, V. et al. Oncogenic mutations in histologically normal endometrium: the new normal? *J. Pathol.* **249**, 173–181 (2019). 31187483 10.1002/path.5314

[63] Anglesio, M. S. et al. Cancer-associated mutations in endometriosis without cancer. *N. Engl. J. Med.* **376**, 1835–1848 (2017). 28489996 10.1056/NEJMoa1614814 PMC5555376

[64] Praetorius, T. H. et al. Molecular analysis suggests oligoclonality and metastasis of endometriosis lesions across anatomically defined subtypes. *Fertil. Steril.* **118**, 524–534 (2022). 35715244 10.1016/j.fertnstert.2022.05.030

[65] Li, L. et al. Genome-wide mutation analysis in precancerous lesions of endometrial carcinoma. *J. Pathol.* **253**, 119–128 (2021). 33016334 10.1002/path.5566

[66] Hu, Z. et al. Proteogenomic insights into early-onset endometrioid endometrial carcinoma: predictors for fertility-sparing therapy response. *Nat. Genet.* **56**, 637–651 (2024). 38565644 10.1038/s41588-024-01703-z

[67] Yizhak, K. et al. RNA sequence analysis reveals macroscopic somatic clonal expansion across normal tissues. *Science* **364**, eaaw0726 (2019). 10.1126/science.aaw0726 PMC7350423 31171663

[68] Zhao, Y. et al. Metformin is associated with reduced cell proliferation in human endometrial cancer by inhibiting PI3K/AKT/mTOR signaling. *Gynecol. Endocrinol.* **34**, 428–432 (2018). 29182407 10.1080/09513590.2017.1409714

[69] Davis, S. R. et al. The benefits of adding metformin to tamoxifen to protect the endometrium—a randomized placebo-controlled trial. *Clin. Endocrinol.* **89**, 605–612 (2018). 10.1111/cen.13830 30107043

[70] Sholl, L. M. et al. Institutional implementation of clinical tumor profiling on an unselected cancer population. *JCI Insight* **1**, e87062 (2016). 27882345 10.1172/jci.insight.87062 PMC5111542

[71] Evans, E. et al. Whole exome sequencing provides loss of heterozygosity (LoH) data comparable to that of whole genome sequencing (171). *Gynecol. Oncol.* **166**, S100 (2022).

[72] Ogobuiro, I. et al. Multiomic characterization reveals a distinct molecular landscape in young-onset pancreatic cancer. *JCO Precis. Oncol.* **7**, e2300152 (2023). 37944072 10.1200/PO.23.00152 PMC10645414

[73] Muquith, M. et al. Tissue-specific thresholds of mutation burden associated with anti-PD-1/L1 therapy benefit and prognosis in microsatellite-stable cancers. *Nat. Cancer* **5**, 1121–1129 (2024). 10.1038/s43018-024-00752-x 38528112

[74] Auwera, Van der, G. A & O’Connor, B. D. *Genomics in the Cloud: Using Docker, GATK, and WDL in Terra* (O’Reilly Media, 2020).

[75] Cibulskis, K. et al. ContEst: estimating cross-contamination of human samples in next-generation sequencing data. *Bioinformatics* **27**, 2601–2602 (2011). 21803805 10.1093/bioinformatics/btr446 PMC3167057

[76] Cibulskis, K. et al. Sensitive detection of somatic point mutations in impure and heterogeneous cancer samples. *Nat. Biotechnol.* **31**, 213–219 (2013). 23396013 10.1038/nbt.2514 PMC3833702

[77] Saunders, C. T. et al. Strelka: accurate somatic small-variant calling from sequenced tumor–normal sample pairs. *Bioinformatics* **28**, 1811–1817 (2012). 22581179 10.1093/bioinformatics/bts271

[78] Ramos, A. H. et al. Oncotator: cancer variant annotation tool. *Hum. Mutat.* **36**, E2423–E2429 (2015). 25703262 10.1002/humu.22771 PMC7350419

[79] Costello, M. et al. Discovery and characterization of artifactual mutations in deep coverage targeted capture sequencing data due to oxidative DNA damage during sample preparation. *Nucleic Acids Res.* **41**, e67 (2013). 23303777 10.1093/nar/gks1443 PMC3616734

[80] Giannakis, M. et al. Genomic correlates of immune-cell infiltrates in colorectal carcinoma. *Cell Rep.* **17**, 1206 (2016). 27760322 10.1016/j.celrep.2016.10.009 PMC5638785

[81] Ellrott, K. et al. Scalable open science approach for mutation calling of tumor exomes using multiple genomic pipelines. *Cell Syst.* **6**, 271–281 (2018). 29596782 10.1016/j.cels.2018.03.002 PMC6075717

[82] Taylor-Weiner, A. et al. DeTiN: overcoming tumor-in-normal contamination. *Nat. Methods* **15**, 531–534 (2018). 29941871 10.1038/s41592-018-0036-9 PMC6528031

[83] Stachler, M. D. et al. Paired exome analysis of Barrett’s esophagus and adenocarcinoma. *Nat. Genet.* **47**, 1047–1055 (2015). 26192918 10.1038/ng.3343 PMC4552571

[84] Thorvaldsdottir, H., Robinson, J. T. & Mesirov, J. P. Integrative Genomics Viewer (IGV): high-performance genomics data visualization and exploration. *Brief. Bioinform.* **14**, 178–192 (2013). 22517427 10.1093/bib/bbs017 PMC3603213

[85] Lawrence, M. S. et al. Discovery and saturation analysis of cancer genes across 21 tumour types. *Nature* **505**, 495–501 (2014). 24390350 10.1038/nature12912 PMC4048962

[86] Benjamini, Y. & Hochberg, Y. Controlling the false discovery rate: a practical and powerful approach to multiple testing. *J. R. Stat. Soc. Ser. B.* **57**, 289–300 (1995).

[87] Gopal, R. K. et al. Widespread chromosomal losses and mitochondrial DNA alterations as genetic drivers in Hürthle cell carcinoma. *Cancer Cell* **34**, 242–255 (2018). 30107175 10.1016/j.ccell.2018.06.013 PMC6121811

[88] Sondka, Z. et al. The COSMIC Cancer Gene Census: describing genetic dysfunction across all human cancers. *Nat. Rev. Cancer* **18**, 696–705 (2018). 30293088 10.1038/s41568-018-0060-1 PMC6450507

[89] Carter, S. L. et al. Absolute quantification of somatic DNA alterations in human cancer. *Nat. Biotechnol.* **30**, 413–421 (2012). 22544022 10.1038/nbt.2203 PMC4383288

[90] Chung, J. et al. DNA polymerase and mismatch repair exert distinct microsatellite instability signatures in normal and malignant human cells. *Cancer Discov.* **11**, 1176–1191 (2021). 33355208 10.1158/2159-8290.CD-20-0790 PMC8223607

[91] Kim, J. et al. Somatic *ERCC2* mutations are associated with a distinct genomic signature in urothelial tumors. *Nat. Genet.* **48**, 600–606 (2016). 27111033 10.1038/ng.3557 PMC4936490

[92] Taylor-Weiner, A. et al. Scaling computational genomics to millions of individuals with GPUs. *Genome Biol.* **20**, 228 (2019). 31675989 10.1186/s13059-019-1836-7 PMC6823959

[93] Haradhvala, N. J. et al. Distinct mutational signatures characterize concurrent loss of polymerase proofreading and mismatch repair. *Nat. Commun.* **9**, 1746 (2018). 29717118 10.1038/s41467-018-04002-4 PMC5931517

[94] Dou, Y. et al. Proteogenomic characterization of endometrial carcinoma. *Cell* **180**, 729–748 (2020). 32059776 10.1016/j.cell.2020.01.026 PMC7233456

[95] Taylor, A. M. et al. Genomic and functional approaches to understanding cancer aneuploidy. *Cancer Cell* **33**, 676–689 (2018). 29622463 10.1016/j.ccell.2018.03.007 PMC6028190

[96] Kuang, Y. et al. Unraveling the clinicopathological features driving the emergence of *ESR1* mutations in metastatic breast cancer. *NPJ Breast Cancer* **4**, 22 (2018). 30083595 10.1038/s41523-018-0075-5 PMC6072793

[97] Janiszewska, M. et al. In situ single-cell analysis identifies heterogeneity for *PIK3CA* mutation and *HER2* amplification in HER2-positive breast cancer. *Nat. Genet.* **47**, 1212–1219 (2015). 26301495 10.1038/ng.3391 PMC4589505

[98] Jung, S. H. Stratified Fisher’s exact test and its sample size calculation. *Biom. J.* **56**, 129–140 (2014). 24395208 10.1002/bimj.201300048 PMC3884832

[99] Martín-Andrés, A. & Herranz-Tejedor, I. Regarding Paper ‘Stratified Fisher’s exact test and its sample size calculation’. *Biom. J.* **57**, 930 (2015). 26059610 10.1002/bimj.201500032

[100] Wu, H. et al. Hypomethylation-linked activation of *PAX2* mediates tamoxifen-stimulated endometrial carcinogenesis. *Nature* **438**, 981–987 (2005). 16355216 10.1038/nature04225

[101] Ritchie, M. E. et al. limma powers differential expression analyses for RNA-sequencing and microarray studies. *Nucleic Acids Res.* **43**, e47 (2015). 25605792 10.1093/nar/gkv007 PMC4402510

[102] Kuleshov, M. V. et al. Enrichr: a comprehensive gene set enrichment analysis web server 2016 update. *Nucleic Acids Res.* **44**, W90–W97 (2016). 27141961 10.1093/nar/gkw377 PMC4987924

[103] Schaefer, C. F. et al. PID: the Pathway Interaction Database. *Nucleic Acids Res.* **37**, D674–D679 (2009). 18832364 10.1093/nar/gkn653 PMC2686461

[104] Reid, J. M. et al. Pharmacokinetics of endoxifen and tamoxifen in female mice: implications for comparative in vivo activity studies. *Cancer Chemother. Pharmacol.* **74**, 1271–1278 (2014). 25318936 10.1007/s00280-014-2605-7 PMC4343319

[105] De Clercq, K., Hennes, A. & Vriens, J. Isolation of mouse endometrial epithelial and stromal cells for in vitro decidualization. *J. Vis. Exp.*10.3791/55168 (2017). 10.3791/55168 PMC5408775 28287563

[106] Cornwell, M. et al. VIPER: Visualization Pipeline for RNA-seq, a Snakemake workflow for efficient and complete RNA-seq analysis. *BMC Bioinformatics* **19**, 135 (2018). 29649993 10.1186/s12859-018-2139-9 PMC5897949

[107] Trapnell, C. et al. Transcript assembly and quantification by RNA-seq reveals unannotated transcripts and isoform switching during cell differentiation. *Nat. Biotechnol.* **28**, 511–515 (2010). 20436464 10.1038/nbt.1621 PMC3146043

[108] Wang, L., Wang, S. & Li, W. RSeQC: quality control of RNA-seq experiments. *Bioinformatics* **28**, 2184–2185 (2012). 22743226 10.1093/bioinformatics/bts356

[109] Love, M. I., Huber, W. & Anders, S. Moderated estimation of fold change and dispersion for RNA-seq data with DESeq2. *Genome Biol.* **15**, 550 (2014). 25516281 10.1186/s13059-014-0550-8 PMC4302049

