# Supplementary material for: Tamoxifen induces PI3K activation in uterine cancer
Source: Nat Genet. 2025 Aug 22;57(9):2192–202. doi: 10.1038/s41588-025-02308-w (PMC12425819; doi:10.1038/s41588-025-02308-w)
Supplement: Supplementary file 1 — Supplementary Figs. 1 and 2 and Notes 1–7. [file 41588_2025_2308_MOESM1_ESM.pdf]

nature genetics

Supplementary information https://doi.org/10.1038/s41588-025-02308-w

Tamoxifen induces PI3K activation in uterine cancer

In the format provided by the authors and unedited

Table of Contents

Supplementary Note 1. Molecular subtypes in TA-UC 2

Supplementary Note 2. Mutational signatures in TA-UC 2

Supplementary Note 3. Analysis of clinicopathological parameters 3

Supplementary Note 4. Analysis of PIK3CA and PIK3R1 coverage 4

Supplementary Note 5. Technical validation confirms low PIK3CA mutation frequency 5

Supplementary Note 6. Effects of estrogen and tamoxifen on PIK3CA 5

Supplementary Note 7. Gene expression analysis reveals pathway differences 6

References 7

Supplementary Note 1. Molecular subtypes in TA-UC

To characterize molecular subtypes in tamoxifen-associated uterine cancer (TA-UC) in more detail, we analyzed the discovery cohort[1] (**Extended Data Fig. 1c**) and identified the following distribution: one (5%) POLE ultra-mutated TA-UC consistent with a *POLE* exonuclease domain mutation (P286R); three (14%) hyper-mutated TA-UCs with microsatellite instability (MSI) in line with their MSIDetect classification (**Extended Data Fig. 1d**); nine (43%) chromosomally instable (CIN) TA-UCs; and eight (38%) genomically stable (GS) TA-UCs.

We further explored potential tamoxifen-driven DNA damage within the molecular subtypes. As expected, the POLE ultra-mutated and MSI hyper-mutated subtypes exhibited the highest number of mutations in both TA-UC and spontaneously arising de novo (i.e., not tamoxifen-associated) UC (all $Q > 0.1$, Wilcoxon test with Benjamini-Hochberg; **Extended Data Fig. 1f**; **Supplementary Table 2**). Likewise, the genomic fractions altered by somatic copy number alterations (SCNAs) were comparable between TA-UC and de novo UC (all $Q > 0.1$; **Extended Data Fig. 1g**; **Supplementary Table 2**), regardless of whether amplified and deleted chromosomal regions were combined or only deleted regions were considered (molecular CIN and GS classes are characterized by a unique deletion-dominated copy number pattern).

Supplementary Note 2. Mutational signatures in TA-UC

To further investigate mutagenic processes active in TA-UC, we examined mutational signatures in the discovery cohort. Our analysis identified the predominant mutational processes previously described in de novo UC[2-4]: POLE signature S1 (cosine similarity 0.71 to COSMIC[5] SBS10b); aging signatures S2 (cosine similarity 0.71 to COSMIC SBS40) and S4 (cosine similarity 0.79 to COSMIC SBS1); and MSI signature S3 (cosine similarity 0.96 to COSMIC

SBS6; **Extended Data Fig. 2a**). We next quantified the activity and relative contribution of these mutational processes across samples. Aging signatures dominated in the majority of cases (17/21), while the POLE (1/21) or MSI (3/21) signatures predominated in only a few cases (**Extended Data Fig. 2b**).

Supplementary Note 3. Analysis of clinicopathological parameters

We evaluated clinicopathological parameters to assess their variability between the TA-UC and de novo UC cohorts and their potential influence on the observed lower *PIK3CA* mutation frequencies in TA-UC.

First, we analyzed the distribution of tumor grades in TA-UC samples from the discovery cohort compared to de novo UC cases from The Cancer Genome Atlas (TCGA) Program (**Supplementary Fig. 1a**). Tumor grade distributions differed significantly between the cohorts for G1 tumors ($Q = 0.004$, Benjamini-Hochberg corrected Fisher's exact test), G3 tumors ($Q = 8\times10^{-7}$) and ungradable tumors ($Q = 0.02$).

We next analyzed population descriptors based on the annotations available in the clinical gene panel sequencing data (**Supplementary Fig. 1b**). In both cohorts, the majority of patients were classified as White, although the proportion of White patients was higher in the de novo UC dataset ($Q = 0.007$) whereas the proportion of patients labeled as other/unknown was higher in the TA-UC dataset ($Q = 3\times10^{-5}$).

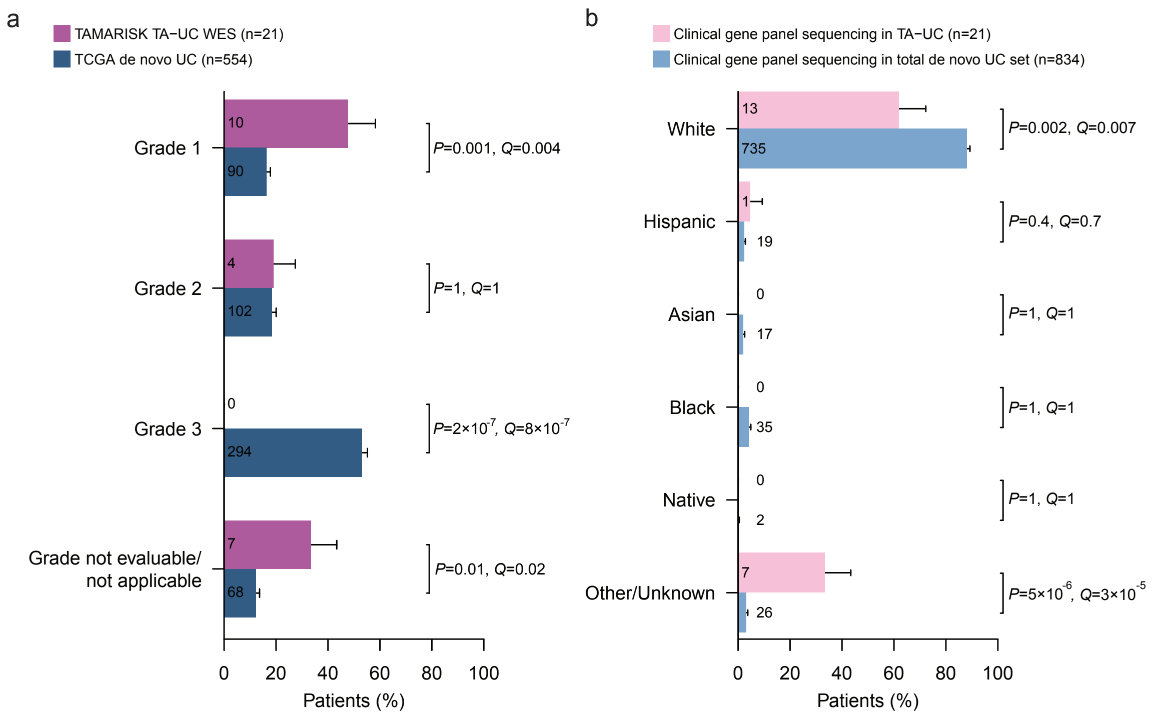

Supplementary Fig. 1. Clinicopathological parameters. (a) Bar plot of cases from de novo UC (TCGA) and the TA-UC discovery cohort (TAMARISK); bars represent tumor grade frequencies. Error bars reflect standard deviation from the β-distribution; significance analysis by two-sided Fisher's exact test with/without Benjamini-Hochberg correction; numbers in bars indicate counts per groups. **(b)** Bar plot of population descriptors, as annotated in the GENIE de novo UC dataset and the clinicogenomic TA-UC validation cohort. Bars represent frequencies; error bars reflect standard deviation from the β-distribution; significance analysis by two-sided Fisher's exact test with/without Benjamini-Hochberg correction; numbers in/above bars indicate counts per group.

Supplementary Note 4. Analysis of *PIK3CA* and *PIK3R1* coverage

As a control for the uneven sequencing coverage of whole-exome sequencing (WES), which may limit our ability to detect *PIK3CA* and *PIK3R1* mutations in poorly covered regions, we calculated the mean sequencing coverage for exonic regions (**Methods**). We then restricted our analysis to TA-UC samples with sufficient mean sequencing coverage for these genes (> 10 reads/bp; **Extended Data Fig. 2e**). Even in this restricted set, *PIK3CA* (8% [1/13] in TA-UC vs 48% [265/554] in TCGA de novo UC, P = 0.004, Fisher's exact test) and *PIK3R1* (0% [0/16] vs 31% [174/554], P = 0.004) remained less frequently mutated in TA-UC. Similar results were found when considering only *PIK3CA* hotspot mutations (8% [1/13] vs 38% [208/554], P = 0.04).

Together, these findings rule out the possibility that the lower mutation frequency observed in *PIK3CA* and *PIK3R1* in TA-UC are due to a technical artifact.

Supplementary Note 5. Technical validation confirms low *PIK3CA* mutation frequency

We validated the presence of *PIK3CA* mutations in our TA-UC discovery cohort using droplet digital PCR (ddPCR) targeting the most common hotspot mutations (E542K, E545K, H1047R). ddPCR experimentally confirmed (**Extended Data Fig. 2f**) the two previously identified *PIK3CA* H1047R mutations and additionally detected an E545K mutation that was missed in WES due to low sequencing coverage (*PIK3CA* mean coverage 1.26x). Even when including this additional mutation, the overall frequency of *PIK3CA* mutations in TA-UC remained significantly lower than in de novo UC ($P = 0.01$). These results confirm the technical accuracy of our findings and reinforce the robustness of the low *PIK3CA* mutation frequency in TA-UC.

Supplementary Note 6. Effects of estrogen and tamoxifen on *PIK3CA*

Since elevated estrogen[6-8] is a known risk factor for the development of de novo UC, we next asked whether high estrogen levels have a similar effect as tamoxifen and whether tumors associated with high estrogen levels also have a lower frequency of *PIK3CA* mutations. Unopposed estrogen administration is no longer used in clinical practice due to its increased risk of UC[9,10] and thus we cannot rule out the possibility that high levels of exogenous estrogen supplementation might also result in a lower frequency of *PIK3CA* mutations, similar to tamoxifen. However, given that obesity is associated with increased levels of endogenous estrogen[11] and higher UC risk[12] , we used obesity status as a proxy for increased estrogen exposure in our analysis. We posited that if relatively higher estrogen levels acted similarly to tamoxifen, we would observe

a lower frequency of *PIK3CA* mutations in obese, but not in normal weight individuals. However, our analysis showed no significant differences in the frequency of *PIK3CA* mutations when comparing de novo UC from normal weight (body mass index [BMI] <25), overweight (BMI 25-29.9), and obese (BMI ≥30) patients in both the TCGA and the Clinical Proteomic Tumor Analysis Consortium (CPTAC) cohorts[13] (all $P \geq 0.1$, Fisher's exact test; **Extended Data Fig. 5**). These results suggest that the lower frequency of *PIK3CA* mutations in TA-UC is due to a mechanism that is different from the effects of higher levels of endogenous estrogen, and that tamoxifen likely has a unique tumorigenic process in the uterus.

Supplementary Note 7. Gene expression analysis reveals pathway differences

As another approach to explore the differences between TA-UC and de novo UC associated with high estragon levels, we performed a differential gene expression analysis using publicly available microarray data from primary human endometrial cells treated in short-term culture with either estradiol (E2) or tamoxifen[14].

This analysis identified 1,760 upregulated genes in response to E2 and 1,745 upregulated genes in response to tamoxifen ($|\log_2(FC)|>\log_2(1.5)$, $Q<0.1$, Benjamini-Hochberg corrected t-test; **Supplementary Fig. 2a**). Albeit small numbers, important differences emerged. In E2-treated cells, genes associated with the estrogen receptor (ER) pathway, including the classical ER-regulated genes *CA12* and *GREB1*, were upregulated, whereas tamoxifen-treated cells showed an upregulation of genes associated with the PI3-AKT-MTOR pathway. These differences were further confirmed by pathway enrichment analysis (**Supplementary Fig. 2b**). We consider these results to be preliminary, as expression data were derived from 1 or 2 individuals per group and

were limited to microarray data. However, even in this small dataset, we found evidence for the activation of the PI3K pathway in TA-UC but not in E2-driven de novo UC.

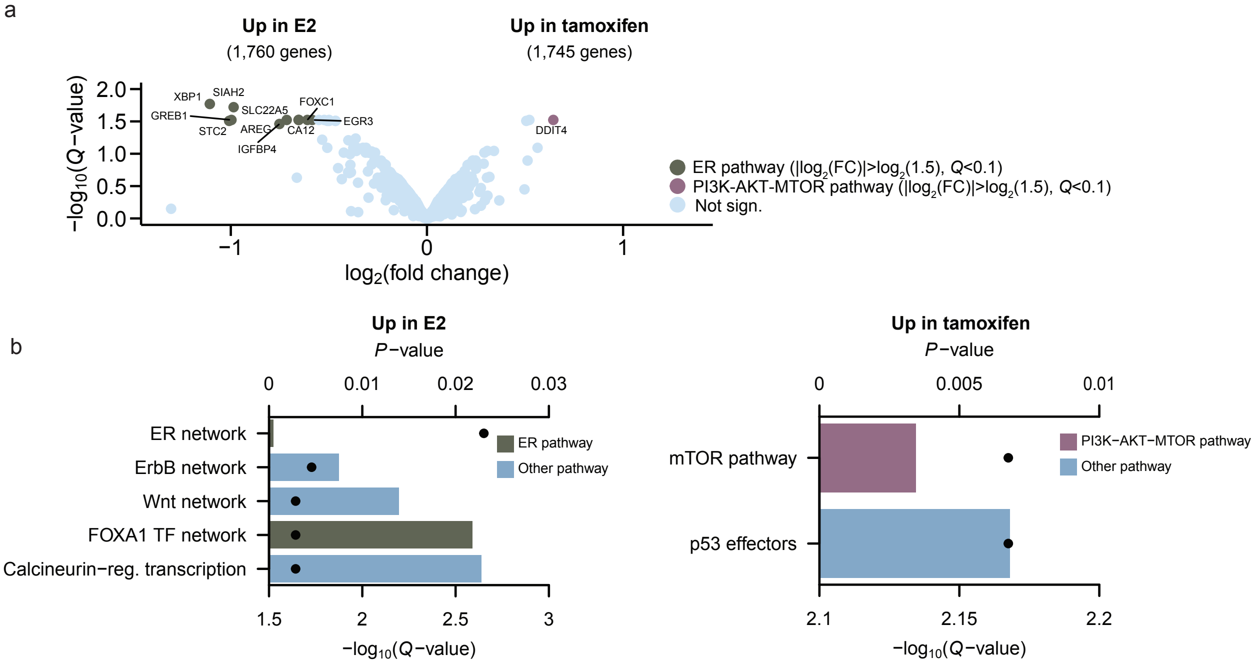

Supplementary Fig. 2. Differential gene expression between E2- and tamoxifen-treated human endometrial cells. (a) Published microarray data from enriched human-derived endometrial cells were quantile normalized and differential expression was tested measuring the effect of tamoxifen using limmaVoom. Volcano plot shows the differentially expressed genes between the E2- (n=1) and tamoxifen-stimulated (n=2) cells. Darker colors represent genes with $|\log_2(FC)|>\log_2(1.5)$ and $Q<0.1$ with dark pink depicting genes from the PI3-AKT pathway and dark green depicting genes from the E2 pathway; Benjamini-Hochberg corrected t-test; total numbers of differentially expressed genes are shown. **(b**) Bar plots depict the *P*-values (all $P<0.05$; $Q<0.1$; Fisher's exact test with Benjamini-Hochberg) of pathway enrichment using Enrichr and NCI pathways in the upregulated genes with $|\log_2(FC)|>\log_2(1.5)$ and $Q<0.1$ from the volcano plot; dots indicate *Q*-values (ER, estrogen receptor, TF, transcription factor; reg, regulated).

References

1. Hoogendoorn, W.E. et al. Prognosis of uterine corpus cancer after tamoxifen treatment for breast cancer. Breast Cancer Res Treat 112, 99-108 (2008).
2. Levine, D.A. et al. Integrated genomic characterization of endometrial carcinoma. Nature 497, 67-73 (2013).
3. Cherniack, A.D. et al. Integrated Molecular Characterization of Uterine Carcinosarcoma. Cancer Cell 31, 411-423 (2017).
4. Cancer Genome Atlas Research Network. Comprehensive and Integrated Genomic Characterization of Adult Soft Tissue Sarcomas. Cell 171, 950-965 e28 (2017).

5. Alexandrov, L.B. et al. The repertoire of mutational signatures in human cancer. Nature 578, 94-101 (2020).
6. Dashti, S.G. et al. Adiposity and breast, endometrial, and colorectal cancer risk in postmenopausal women: Quantification of the mediating effects of leptin, C-reactive protein, fasting insulin, and estradiol. Cancer Med 11, 1145-1159 (2022).
7. Kaaks, R., Lukanova, A. & Kurzer, M.S. Obesity, endogenous hormones, and endometrial cancer risk: a synthetic review. Cancer Epidemiol Biomarkers Prev 11, 1531-43 (2002).
8. Schmandt, R.E., Iglesias, D.A., Co, N.N. & Lu, K.H. Understanding obesity and endometrial cancer risk: opportunities for prevention. Am J Obstet Gynecol 205, 518-25 (2011).
9. Smith, D.C., Prentice, R., Thompson, D.J. & Herrmann, W.L. Association of exogenous estrogen and endometrial carcinoma. N Engl J Med 293, 1164-7 (1975).
10. Ziel, H.K. & Finkle, W.D. Increased risk of endometrial carcinoma among users of conjugated estrogens. N Engl J Med 293, 1167-70 (1975).
11. Freeman, E.W., Sammel, M.D., Lin, H. & Gracia, C.R. Obesity and reproductive hormone levels in the transition to menopause. Menopause 17, 718-26 (2010).
12. Onstad, M.A., Schmandt, R.E. & Lu, K.H. Addressing the Role of Obesity in Endometrial Cancer Risk, Prevention, and Treatment. J Clin Oncol 34, 4225-4230 (2016).
13. Dou, Y. et al. Proteogenomic Characterization of Endometrial Carcinoma. Cell 180, 729-748 e26 (2020).
14. Wu, H. et al. Hypomethylation-linked activation of PAX2 mediates tamoxifen-stimulated endometrial carcinogenesis. Nature 438, 981-7 (2005).
