# Supplementary material for: Tamoxifen induces PI3K activation in uterine cancer
Source: Nat Genet. 2025 Aug 22;57(9):2192–202. doi: 10.1038/s41588-025-02308-w (PMC12425819; doi:10.1038/s41588-025-02308-w)
Supplement: Supplementary file 2 — Reporting Summary [file 41588_2025_2308_MOESM2_ESM.pdf]

nature portfolio

Corresponding author(s): Gad Getz, Rinath Jeselsohn

Last updated by author(s): Apr 22, 2025

Reporting Summary

Nature Portfolio wishes to improve the reproducibility of the work that we publish. This form provides structure for consistency and transparency in reporting. For further information on Nature Portfolio policies, see our Editorial Policies and the Editorial Policy Checklist.

Statistics

For all statistical analyses, confirm that the following items are present in the figure legend, table legend, main text, or Methods section.

n/a	Confirmed	
☐	☒	The exact sample size (*n*) for each experimental group/condition, given as a discrete number and unit of measurement
☐	☒	A statement on whether measurements were taken from distinct samples or whether the same sample was measured repeatedly
☐	☒	The statistical test(s) used AND whether they are one- or two-sided<br>*Only common tests should be described solely by name; describe more complex techniques in the Methods section.*
☐	☒	A description of all covariates tested
☐	☒	A description of any assumptions or corrections, such as tests of normality and adjustment for multiple comparisons
☐	☒	A full description of the statistical parameters including central tendency (e.g. means) or other basic estimates (e.g. regression coefficient) AND variation (e.g. standard deviation) or associated estimates of uncertainty (e.g. confidence intervals)
☐	☒	For null hypothesis testing, the test statistic (e.g. *F*, *t*, *r*) with confidence intervals, effect sizes, degrees of freedom and *P* value noted<br>*Give P values as exact values whenever suitable.*
☐	☒	For Bayesian analysis, information on the choice of priors and Markov chain Monte Carlo settings
☒	☐	For hierarchical and complex designs, identification of the appropriate level for tests and full reporting of outcomes
☐	☒	Estimates of effect sizes (e.g. Cohen's *d*, Pearson's *r*), indicating how they were calculated

Our web collection on statistics for biologists contains articles on many of the points above.

Software and code

Policy information about availability of computer code

Data collection	Leica Application Suite X (LAS X, v3.7), QuPath (v0.2.0)
Data analysis	'R' (v4.1.1) in an RStudio environment, Julia (v1.7.3) in a Jupyter environment, Picard Tools data processing pipeline (v2.9.2), ContEst (v4), DepthOfCoverage function from GATK (v4.1.6.0.), MuTect (v1), Strelka (v2.9.0), Oncotator (v1.9.0.0), OxoG / FFPE / PoN filters (v25), deTiN (v3.0), 'force-calling' method (v2), IGV (v2.16.2), MutSig2CV (v3.11), GATK4's Copy Number Variant (CNV) discovery pipeline (v4.1.6.0), GISTIC2.0 (v2.03.23), ABSOLUTE (v1.5), PhylogicNDT (v35), MSIDetect (v2), SignatureAnalyzer (v0.0.8), GTEx pipeline (v8), limmaVoom (v3.50.0), GraphPad Prism (v9.0), VIPER (v1.41.0), STAR (v2.7.0f), cufflinks (v2.2.1), RSeQC (v2.6.2), DEseq2 (v1.18.1), Enrichr (https://maayanlab.cloud/Enrichr/). All software packages and code are publicly available, and their sources are cited in the Methods section.

For manuscripts utilizing custom algorithms or software that are central to the research but not yet described in published literature, software must be made available to editors and reviewers. We strongly encourage code deposition in a community repository (e.g. GitHub). See the Nature Portfolio guidelines for submitting code & software for further information.

Data

Policy information about availability of data

All manuscripts must include a data availability statement. This statement should provide the following information, where applicable:

- Accession codes, unique identifiers, or web links for publicly available datasets
- A description of any restrictions on data availability
- For clinical datasets or third party data, please ensure that the statement adheres to our policy

TCGA pancancer data are available through a data portal: https://gdc.cancer.gov/node/905/; https://gdc.cancer.gov/about-data/publications/pancanatlas. In compliance with the data access policy, most data are in an open tier that does not require access approval. Some data files with potentially identifying information and underlying sequencing data are controlled-access data and may be hosted at dbGaP. Researchers will need to apply to the TCGA Data Access Committee (DAC) via dbGaP (https://dbgap.ncbi.nlm.nih.gov/aa/wga.cgi?page = login) to request access. CPTAC endometrial cancer mutation data are available from the Genomic Data Commons (https://gdc.cancer.gov/) or upon request from dbGaP (https://www.ncbi.nlm.nih.gov/gap/, phs001287). SEER data are available through a data portal (https://seer.cancer.gov/data/) after data use agreement forms have been signed. The Affymetrix U95A Human Genome arrays of enriched human-derived endometrial cells can be accessed via GSE3013. Data from the GENIE database can be found on the Sage Bionetworks portal (https://www.synapse.org/#!Synapse:syn7222066/wiki/405659). To request access to protected GENIE data, researchers need to apply to dbGaP for access (study accession phs001337). Analyses in this paper also used published data sets that are available from the corresponding manuscripts, which are referenced where relevant. The whole exome sequencing data of TA-UCs is available through EGA, the accession number is EGAS00001006453 (https://www.ega-archive.org/studies/EGAS00001006453). The mouse endometrial epithelial RNAseq data is available through GSE179647 (https://www.ncbi.nlm.nih.gov/geo/query/acc.cgi?acc=GSE179647). The Caris datasets generated during and/or analyzed during the current study are available from the corresponding author on reasonable request. The deidentified sequencing data are owned by Caris Life Sciences. Qualified researchers can apply for access to these summarized data by contacting the corresponding author. MsigDB oncogenic signatures, KEGG pathway database, estrogen response genes from the hallmark gene sets and genes in the AKT/MTOR oncogenic signature gene sets are from: https://www.gsea-msigdb.org/gsea; the NCI-Nature Pathway Interaction Database can be found at: https://www.ndexbio.org/.

Research involving human participants, their data, or biological material

Policy information about studies with human participants or human data. See also policy information about sex, gender (identity/presentation), and sexual orientation and race, ethnicity and racism.

Reporting on sex and gender	This study uses a dataset of women diagnosed with uterine cancer during/after tamoxifen treatment.
Reporting on race, ethnicity, or other socially relevant groupings	Ethnicity data were available for the following cohorts: (i) TCGA de novo uterine cancer cases, which included White (70%), Asian (4%), Black (16%), Hispanic (3%), Native (2%), and unknown/other (5%); (ii) GENIE de novo uterine cancer cases, comprising White (88%), Asian (2%), Black (4%), Hispanic (2%), Native (1%), and unknown/other (3%); and (iii) GENIE tamoxifen-associated uterine cancer cases, consisting of White (62%), Hispanic (5%), and unknown/other (33%).
Population characteristics	Clinical data are provided in Supplementary Table 1. The median age of women diagnosed with tamoxifen-associated uterine cancer (TA-UC) across all cohorts ranged from 66 to 74 years. In the discovery cohort, the median age was 73 years (range: 50-90) and in the validation cohorts 74 years (range: 46-85) and 58 years (range: 44-72), respectively. Across all cohorts, 47% of women (60/128) were diagnosed with uterine cancer during tamoxifen treatment or within the first year after stopping tamoxifen ('users'), and 53% (68/128) were diagnosed the first year after stopping tamoxifen use ('ex-users').
Recruitment	Patients were recruited to the retrospective cohort study TAMARISK (Tamoxifen Associated Malignancies: Aspects of Risk) by the participating Comprehensive Cancer Center of the TAMARISK group, following local protocols. Additional data were identified from the following clinical databases: (i) through cancer registry data at DFCI by cross-referencing uterine cancer diagnoses with breast cancer and tamoxifen treatment to find cases with uterine cancer genotype data; and (ii) using the Caris Life Sciences-internal instance of cBioPortal to search for patients treated with tamoxifen for breast cancer who were later diagnosed with uterine cancer.
Ethics oversight	Samples were collected and sequenced with the approval of the Institutional Review Board of the The Netherlands Cancer Institute, Amsterdam (protocol #CFMPB294) and DFCI (protocol #12-049B). Approval for the clinical data from DFCI was granted under protocols #17-000 and #11-104. In accordance with 45 CFR 46.101(b), the retrospective study of de-identified clinical data from Caris Life Sciences was deemed exempt by the Institutional Review Board, and patient consent was not required.

Note that full information on the approval of the study protocol must also be provided in the manuscript.

Field-specific reporting

Please select the one below that is the best fit for your research. If you are not sure, read the appropriate sections before making your selection.

☒ Life sciences ☐ Behavioural & social sciences ☐ Ecological, evolutionary & environmental sciences

For a reference copy of the document with all sections, see nature.com/documents/nr-reporting-summary-flat.pdf

Life sciences study design

All studies must disclose on these points even when the disclosure is negative.

Sample size	The TAMARISK discovery cohort consisted of 21 samples; the TAMARISK validation cohort consisted of 39 samples; the clinical gene panel sequencing validation cohort consisted of 21 samples; and the clinical WES sequencing validation cohort consisted of 47 samples.<br>No formal sample size calculation was performed upfront. However, the discovery cohort (n = 21) was considered sufficient for exploratory genomic analysis based on the observed mutation burden in TA-UC of median of 2.7 mutations/Mb. This sample size provides 5% power to detect genes mutated in at least 10% of patients. For validation, we analyzed an independent cohort of 47 patients, which provided 81% power to detect genes mutated in 20% of patients at the same mutation rate.
Data exclusions	No data was excluded from the analysis.
Replication	The findings of this study from the TAMARISK discovery cohort were validated in three independent cohorts: (i) 39 TA-UC were subjected to droplet digital PCR (ddPCR) and constitute the TAMARISK validation cohort (Extended Data Fig. 1a, Extended Data Fig. 6a-b, Supplementary Table 1); (ii) 21 TA-UC were identified from a cohort subjected to clinical gene panel sequencing (Fig. 2a, Extended Data Fig. 6c-e, Supplementary Tables 1 & 8); and (iii) 47 TA-UC were identified from a cohort subjected to clinical WES sequencing (Fig. 2b, Extended Data Fig. 6f-j, Supplementary Table 1).
Randomization	This is a retrospective study, and randomization was not possible.
Blinding	No blinding was undertaken as this was a study involving genomic analyses using predefined computational pipelines, which are not subject to bias.

Reporting for specific materials, systems and methods

We require information from authors about some types of materials, experimental systems and methods used in many studies. Here, indicate whether each material, system or method listed is relevant to your study. If you are not sure if a list item applies to your research, read the appropriate section before selecting a response.

Materials & experimental systems

n/a	Involved in the study	
☐	☒	Antibodies
☒	☐	Eukaryotic cell lines
☒	☐	Palaeontology and archaeology
☐	☒	Animals and other organisms
☒	☐	Clinical data
☒	☐	Dual use research of concern
☒	☐	Plants

Methods

n/a	Involved in the study	
☒	☐	ChIP-seq
☒	☐	Flow cytometry
☒	☐	MRI-based neuroimaging

Antibodies

Antibodies used	anti-mouse (G21040, 1:2000, Invitrogen)<br>anti-rabbit (G21234, 1:2000, Invitrogen)<br>ER-alpha (06-938, 1:1000, Millipore)<br>Phospho-IR/IGF1R Tyr1162-3 (44-804, 1:500; Invitrogen)<br>Ki67 (ab15580, 1:1000; Abcam)<br>phospho-AKT Thr308 (ab81283, 1:50; Abcam)<br>phospho-S6 Ser240/244 (2215, 1:500; Cell signaling)
Validation	The above antibodies are commercially available and have been validated by the manufacturers for immunohistochemistry in mouse tissues (see the manufacturers' websites for details).

Animals and other research organisms

Policy information about studies involving animals; ARRIVE guidelines recommended for reporting animal research, and Sex and Gender in Research

Laboratory animals	C57BL/6 female mice (Jackson Laboratories), aged 6-7 weeks (after puberty). Mice were housed as up to 5 mice/cage. The room temperature was kept at 20 – 26 °C and humidity 30 – 70%. The animals were under a 12 h:12 h light-dark cycle and had ad libitum access to water and irradiated Laboratory Rodent Diet. Animals had at least 3 days of acclimation prior to study initiation.
Wild animals	The study did not involve wild animals.

Reporting on sex	Only female mice were used in this study, as the overall objective was to evaluate the effect of tamoxifen on uterine cancer development.
Field-collected samples	The study did not involve samples collected from the field.
Ethics oversight	All mice were maintained in accordance with local guidelines, and therapeutic interventions were approved by the Animal Care and Use Committees of Dana-Farber Cancer Institute (DFCI).

Note that full information on the approval of the study protocol must also be provided in the manuscript.

Plants

Seed stocks	*Report on the source of all seed stocks or other plant material used. If applicable, state the seed stock centre and catalogue number. If plant specimens were collected from the field, describe the collection location, date and sampling procedures.*
Novel plant genotypes	*Describe the methods by which all novel plant genotypes were produced. This includes those generated by transgenic approaches, gene editing, chemical/radiation-based mutagenesis and hybridization. For transgenic lines, describe the transformation method, the number of independent lines analyzed and the generation upon which experiments were performed. For gene-edited lines, describe the editor used, the endogenous sequence targeted for editing, the targeting guide RNA sequence (if applicable) and how the editor was applied.*
Authentication	*Describe any authentication procedures for each seed stock used or novel genotype generated. Describe any experiments used to assess the effect of a mutation and, where applicable, how potential secondary effects (e.g. second site T-DNA insertions, mosiacism, off-target gene editing) were examined.*
